# ParB proteins can bypass DNA-bound roadblocks via dimer-dimer recruitment

**DOI:** 10.1126/sciadv.abn3299

**Published:** 2022-06-29

**Authors:** Miloš Tišma, Maria Panoukidou, Hammam Antar, Young-Min Soh, Roman Barth, Biswajit Pradhan, Anders Barth, Jaco van der Torre, Davide Michieletto, Stephan Gruber, Cees Dekker

**Affiliations:** 1 Department of Bionanoscience, Kavli Institute of Nanoscience Delft, Delft University of Technology, Delft, Netherlands.; 2 School of Physics and Astronomy, University of Edinburgh, Edinburgh, UK.; 3 Department of Fundamental Microbiology (DMF), Faculty of Biology and Medicine (FBM), University of Lausanne (UNIL), Lausanne, Switzerland.; 4 MRC Human Genetics Unit, Institute of Genetics and Cancer, University of Edinburgh, Edinburgh, UK.

## Abstract

The ParAB*S* system is essential for prokaryotic chromosome segregation. After loading at *parS* on the genome, ParB (partition protein B) proteins rapidly redistribute to distances of ~15 kilobases from the loading site. It has remained puzzling how this large-distance spreading can occur along DNA loaded with hundreds of proteins. Using in vitro single-molecule fluorescence imaging, we show that ParB from *Bacillus subtilis* can load onto DNA distantly of *parS*, as loaded ParB molecules themselves are found to be able to recruit additional ParB proteins from bulk. Notably, this recruitment can occur in cis but also in trans, where, at low tensions within the DNA, newly recruited ParB can bypass roadblocks as it gets loaded to spatially proximal but genomically distant DNA regions. The data are supported by molecular dynamics simulations, which show that cooperative ParB-ParB recruitment can enhance spreading. *ParS*-independent recruitment explains how ParB can cover substantial genomic distance during chromosome segregation, which is vital for the bacterial cell cycle.

## Teaser

ParB proteins can recruit other ParB dimers, allowing them to overcome DNA roadblocks and increase spreading distances along DNA.

## INTRODUCTION

Accurate chromosome segregation is crucial for a stable transmission of genetic material through each cell cycle. To actively segregate origins of replication, most prokaryotes rely on the ParAB*S* system (*1*, *2*), which consists of a *parS* binding sequence, the adenosine triphosphatase partition protein A (ParA), and the cytidine triphosphatase (CTPase) ParB (*3*–*6*). ParB dimers bind to the *parS* sequence, located near the origin of replication, spread laterally, and ultimately form ParB-DNA partition complexes that are imperative for DNA segregation (*7*–*13*). A ParA gradient along the cell axis subsequently segregates these complexes to effectively administer the nascent genomes to the daughter cells (*14*, *15*). Proper partitioning of the ParB complexes is vital for bacterial cell survival and has been studied intensively in recent years (*16*), especially since it was recently found that ParB proteins from several model organisms use cytidine 5′-triphosphate (CTP) to load onto a *parS* sequence (*4*, *5*, *17*).

A necessary feature for correct chromosome partitioning comes from a particular ability of ParB proteins to clamp the *parS*-DNA as a dimer (*4*, *5*) and to subsequently slide along the DNA by diffusion, effectively freeing the *parS* site for new ParB proteins to load. ParB has been found to laterally spread over large genomic regions surrounding the *parS* sites in vivo (10 to 15 kb) (*18*–*22*), which was reported to be essential for partition complex formation (*9*, *10*, *16*, *18*, *19*, *23*–*25*). However, in vitro studies showed that single DNA-bound proteins block the diffusion of ParB along DNA very efficiently (*4*, *7*, *17*), raising the question how spreading can occur in a dense cellular environment, where, with ~1 gene and ~20 to 50 nucleoid-associated proteins/kb (*26*, *27*), sliding ParB dimers will continuously run into “roadblocks” that will stall their movement. Theoretical modeling of a ParB “clamping and sliding” model indicated that such roadblocks markedly limit the spreading distance on F plasmids (*28*). However, in vivo chromatin immunoprecipitation sequencing (ChIP-seq) data do not show strong changes in the ParB occupancy in the vicinity of genes and operons (*18*–*22*, *29*).

## RESULTS

**Fig. 1.**
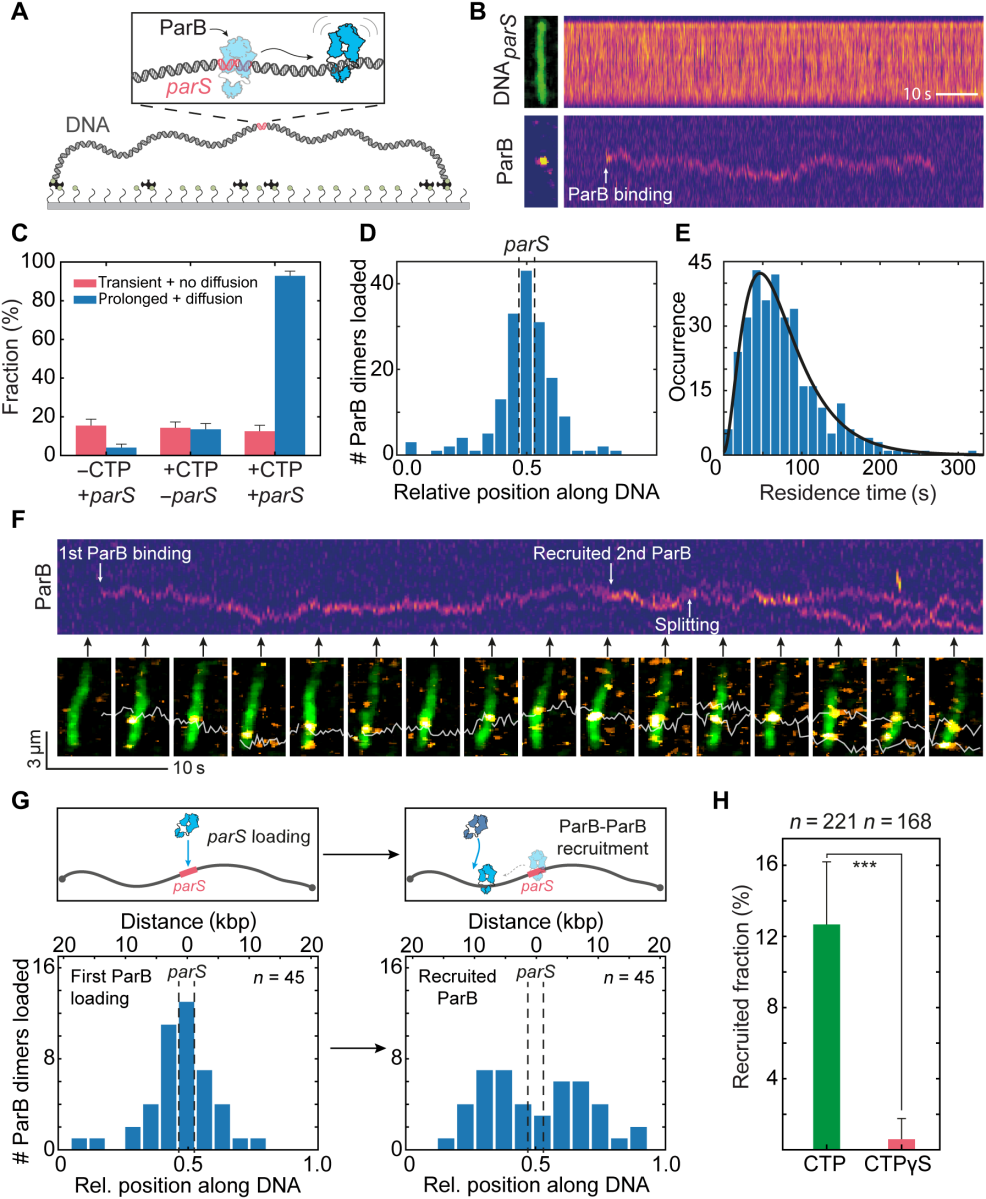
DNA-bound ParB dimers can recruit new ParB dimers independently of *parS*. (**A**) Schematic representation of DNA*_parS_* that is tethered at both its ends to a surface (see also fig. S1). (**B**) Kymographs for DNA*_parS_* stained with SYTOX Green (SxG) (top) and ParB^alexa647^ (bottom). Left images show single-frame snapshots of the DNA and ParB at the moment of binding. White arrow indicates ParB loading. (**C**) Fraction of DNA tethers with transient binding with no diffusion and the fraction showing prolonged binding with diffusion of ParB molecules for various conditions (*n* = 123, 133, and 112 from left to right). Error bars represent binomial proportion confidence intervals. (**D**) Histogram showing loading position of ParB dimers along DNA*_parS_*. The position of *parS* site is represented by dashed lines (*n* = 168). (**E**) Residence times of diffusing ParB dimer molecules after binding DNA*_parS_* (*n* = 332). The data were fitted to a model assuming a delayed dissociation of ParB from the DNA after CTP hydrolysis (black line; see Materials and Methods and fig. S3, B and C). (**F**) Top: Kymograph for ParB^alexa647^. White arrows indicate first ParB-dimer binding, second ParB-dimer binding, and splitting. Bottom: Snapshot images, taken at time points indicated by black arrows. White line indicates the single-particle tracking trace. (**G**) Distributions of ParB dimer loading positions. Left: First ParB dimer loading at *parS* site. Right: Loading position of the recruited second ParB dimer. Top panels are illustrative schemes. (**H**) Recruitment rate in presence of CTP (green) or cytidine 5′-(γ-thio)triphosphate (CTPγS) (red). Error bars represent binominal confidence intervals. Statistical significance calculated using chi-square test for a binomial distribution, χ^2^ (*n* = 389) = 20.196; ***P < 0.0001.

Here, we examine the mechanism of ParB spreading using a controlled DNA stretching assay that allows in vitro visualization at the single-molecule level (*30*). First, we verified that CTP and the *parS* site are essential for the loading and diffusion of *Bacillus subtilis* ParB on the DNA. We tethered 42-kb-long DNA molecules with a single *parS* site close to the middle (DNA*_parS_*) to a streptavidin-functionalized surface via their 5′-biotin ends (Fig. 1A and fig. S1A). Highly inclined and laminated optical sheet (HiLo) microscopy was used to observe DNA and proteins labeled with different fluorescent dyes (see fig. S1, B to F).

### CTP hydrolysis determines residence of ParB on DNA

ParB was found to exhibit two different types of behaviors, viz., transient nondiffusive binding at various locations (fig. S2, A to C) and binding at the *parS* site followed by diffusion (Fig. 1B and fig. S2, D and E). Transient binding was short (~5 to 20 s), independent of the presence of CTP in the medium, and only weakly correlated to the *parS* sequence (Fig. 1C and fig. S2, B and C). In the presence of both the *parS* sequence and CTP, however, binding initiated predominantly at the location of the *parS* sequence (Fig. 1D and fig. S2, D to H). Both the transient binding and binding at the *parS* site were dependent on the C-terminal domain, and the previously described mutant (ParB^KKK^ (*31*); see Materials and Methods) that is disabled in nonspecific DNA binding did not show either of these binding types in our fluorescence assay and in a biolayer interferometry assay (fig. S2I) (*4*, *19*, *32*). Unexpectedly, the ParB^KKK^ mutant still showed DNA*_parS_*-stimulated CTP hydrolysis, despite its poor DNA binding (fig. S2J).

When ParB dimers were specifically loaded at *parS* site, they remained bound to the DNA for an average time of 76 ± 2 s (average ± SEM; Fig. 1E), during which time the ParB diffused away from the *parS* site. Taking into account slight effects of the dyes on the CTP hydrolysis rate (fig. S1F), this residence time is in line with the previously determined CTP hydrolysis rate of 1 CTP/100 s for ParB*_BSu_* (*4*), where the hydrolysis of CTP would cause the ParB clamp to destabilize and open, allowing dissociation from the DNA (*4*, *5*, *19*). We observed a nonexponential distribution of ParB residence times, indicating that CTP hydrolysis is rate limiting for dissociation (fig. S3). A model assuming immediate dissociation of ParB after the independent hydrolysis of the two CTP molecules was unable to fully describe the data (see Materials and Methods; fig. S3, B and C; and table S2), suggesting that ParB may remain weakly bound to the DNA after CTP hydrolysis. Accounting for a delayed dissociation of ParB provided a substantially improved description of the experimental data with an average dwell time of ~15 s after CTP hydrolysis (Fig. 1E; fig. S3, B and C; and table S2). The delayed dissociation may originate from nonspecific interaction of the open ParB clamp with the DNA via its positive residue patch at the C terminus (*31*, *33*).

### Diffusing ParB proteins can recruit new dimers onto the DNA

**Fig. 2.**
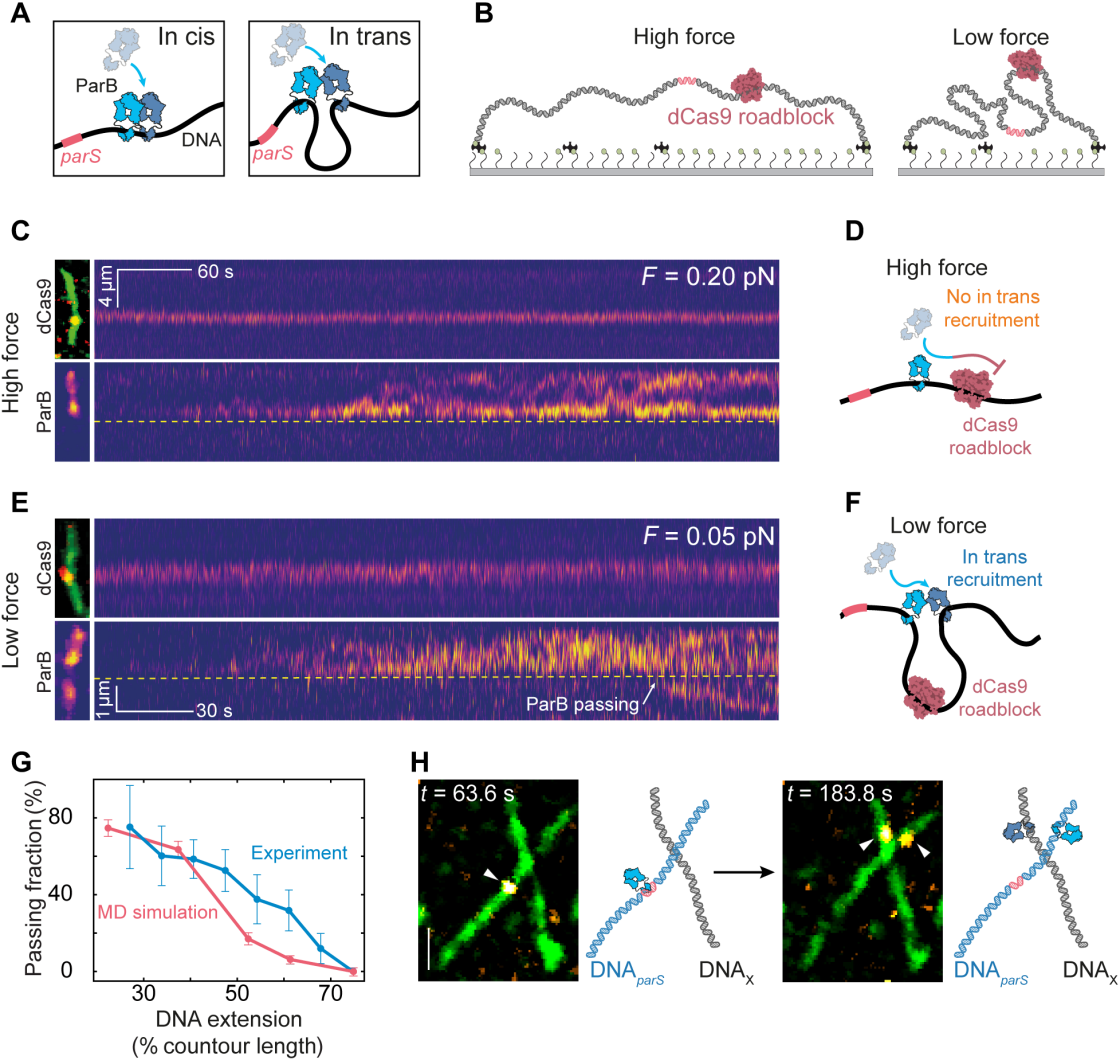
ParB can bypass roadblocks by in trans ParB-ParB recruitment. (**A**) Illustration of in cis (left) and in trans (right) recruitment events. (**B**) Schematic of the roadblock experiment for a large (left; high force) and small DNA*_parS_* end-to-end distance (right; low force). (**C**) Kymograph for dCas9^alexa549^ (top) and ParB^alexa647^ (bottom) for DNA*_parS_* at 0.20 pN. Snapshots of dCas9^alexa549^/^SYTOX Green^DNA*_parS_* overlay and ParB^alexa647^ signal are provided on the left. Yellow dashed line indicates the position of the dCas9^alexa549^. (**D**) Cartoon of the DNA*_parS_* at high force where ParB molecules cannot be recruited across the roadblock. (**E**) Same as (C) but data at a force of 0.05 pN. White arrow denotes a passing event. (**F**) Same as (D) but for low force where ParB molecules can be recruited in trans across the dCas9 roadblock. (**G**) Fraction of DNA molecules that exhibit ParB dimers passing the dCas9 roadblock displayed versus stretching length of the DNA. Error bars represent binomial proportion confidence intervals (blue; *n* = 122) and SD from simulation cycles (pink; *n* = 128). MD, molecular dynamics. (**H**) Left: Snapshot of the DNA*_parS_*-DNA_X_ crossing at the time of the first ParB binding to *parS* site (*t* = 105 s). Right: Snapshot of the same field of view at time *t* = 306 s, where a second ParB was recruited onto the DNA_X_ by in trans recruitment. White arrowheads indicate the positions of ParB dimers. Cartoon representations are provided on the right of the snapshot frames. Scale bar, 2 μm.

Notably, the data also revealed an unexpected behavior that was different from mere loading and diffusion of ParB dimers: A ParB dimer that was previously loaded onto the DNA*_parS_* was observed to be able to load a new ParB dimer onto the DNA (Fig. 1, F and G, and fig. S4, A to E). This behavior was characterized by the recruitment of a second ParB dimer at the site of an existing one (as evidenced from an increase of the fluorescent intensity), a brief colocalization, and a conjunct diffusion of the two ParB dimers (for 8.2 ± 3.5 s, average ± SD; fig. S4D), after which the two dimers split into two independently diffusing ParB dimers (fig. S4, A to C). This intriguing behavior occurred in about 12% of the observed ParB molecules that loaded onto the *parS* site (Fig. 1H), which is significantly higher than accidental colocalization by off-site loading (fig. S4D). We refer to this type of loading behavior as “ParB-ParB recruitment.” We can distinguish ParB-ParB recruitment from simple subsequent loading of ParB proteins at the *parS* site by evaluating the position along DNA where events occur. As expected, the first ParB dimer loaded at the *parS* site near the middle of the DNA*_parS_* (Fig. 1, F and G, left), but the loading of the second recruited ParB dimer occurred later at a position that was, on average, 5.2 ± 3.8 kb (average ± SD, *n* = 46) away from the *parS* site (Fig. 1, F and G, right). Accordingly, the first ParB loaded at *parS* site and already diffused a significant distance before recruiting a new ParB dimer at a distant location (Fig. 1G, right). After ParB-ParB recruitment, we observed that both ParB dimers resided on the DNA for ~72 s, i.e., for a similar amount of time as single diffusing ParB before recruitment (~66 s; fig. S4C). The recruitment thus approximately doubled the residence time of the initially loaded ParB dimer to ~138 s (fig. S4C). Notably, the ParB-ParB recruitment process was highly dependent on the presence of CTP and occurred significantly less at lower CTP concentrations (see below) and hardly at all in the presence of the slowly hydrolysable nucleotide variant cytidine 5′-(γ-thio)triphosphate (CTPγS) (Fig. 1H) (*25*). Hereafter, we refer to these recruitment events where the second ParB loaded at a DNA position adjacent to the first one, as “in cis” (Fig. 2A, left), akin to what was previously proposed for ParB-bridges (*10*).

### In trans ParB-ParB recruitment can overcome DNA-bound roadblocks

Interestingly, we observed that ParB-ParB recruitment events can also happen “in trans,” where the second ParB got loaded onto a DNA position that is genomically distant but transiently proximal in a three-dimensional (3D) space via DNA looping (Fig. 2A, right). Because these types of in trans events could potentially allow for the passage of DNA-bound roadblocks in the crowded in vivo environment, we designed an experiment to test for in trans recruitment in the presence of DNA-bound roadblocks. We placed a firmly bound dCas9 protein on one side of the *parS* sequence on the DNA (Fig. 2B) and tuned the end-to-end distance of the DNA to 65% of its total contour length (corresponding to a stretching force of 0.20 pN within the DNA) (*34*). Under these conditions, we observed that the dCas9 roadblock efficiently prevented the diffusion of ParB proteins (Fig. 2, C and D, and fig. S5, A to E), in line with findings with other DNA-bound roadblocks (*4*, *7*, *17*). However, when repeating the same experiment for a lower end-to-end distance (27% of the DNA*_parS_* contour length; *F =* 0.05 pN) (*34*), we observed events where ParB proteins would cross the roadblock and continue 1D diffusion along the DNA on the other side of dCas9 (Fig. 2, E and F; fig. S6, A to E; and movie S1). We attribute this behavior to the in trans ParB-ParB recruitment. The bypassing of the Cas9 roadblock was also visibly apparent in the cumulated ParB fluorescence intensity signal, which rapidly increased on the *parS* side, while the non-*parS* side displayed discrete increases in intensity only for the nonstretched DNA*_parS_* (figs. S5, C and E, and S6, B and D).

The tension within the DNA appeared to have a significant effect on the success rate of crossing the roadblock. As Fig. 2G displays, the fraction of DNA molecules where ParB successfully passed the dCas9 roadblock decreased from ~80% at 0.05 pN (27% of the total DNA contour length) to 0% at 0.35 pN (75% contour length) (*34*). These data are consistent with in trans ParB-ParB recruitment, where at low stretching forces, regions that are distant in DNA sequence can come into physical contact through bending and looping of the DNA via thermal fluctuations. We further corroborated these findings by observing the ParB-mediated in trans loading of a new ParB dimer onto a different DNA molecule that was spatially nearby, where this DNA did not contain an endogenous *parS* site (Fig. 2H and fig. S7). Here, as previously described, we bound the DNA*_parS_* to the surface but then subsequently bound a new DNA molecule lacking the *parS* site (DNA_X_), perpendicular to the originally bound DNA*_parS_* (fig. S7, A and B). In this assay, we observed events where ParB first specifically bound the DNA*_parS_*, then reached the crossing point by random diffusion, where it recruited a new ParB molecule onto the DNA_X_ (fig. S7C, dashed line), yielding a transfer and subsequent independent 1D diffusion of the newly recruited ParB dimer on the DNA_X_ molecule (Fig. 2H and fig. S7C).

**Fig. 3.**
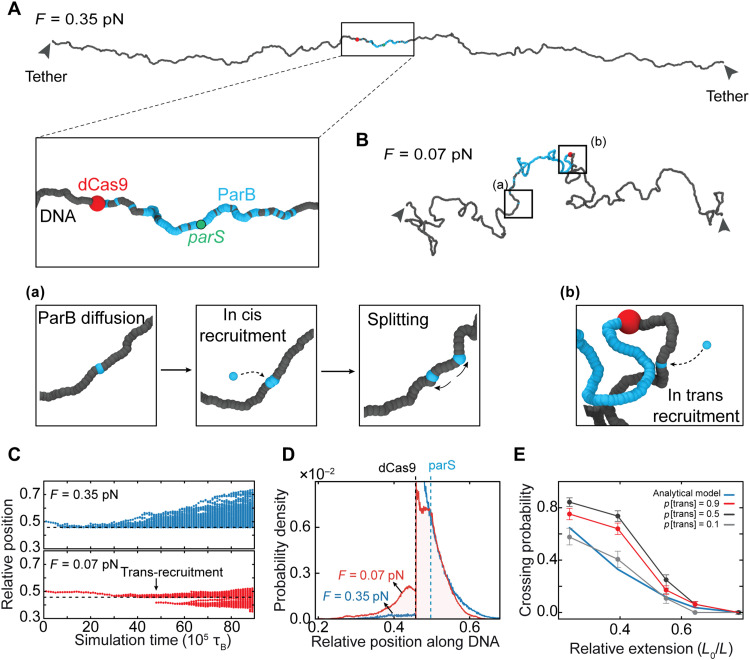
DNA looping via thermal fluctuations allows in trans ParB-ParB recruitment and spreading across roadblocks. (**A**) Single-frame snapshot of the DNA tethers in the molecular dynamics simulations. DNA tether end-to-end length of 11 μm (i.e., 75% contour length and a force of 0.35 pN). Zoomed region highlights the region proximal to *parS* site. Tether has a *parS* site in the middle (green) and simulated dCas9 roadblock on one side (red). ParB represented in yellow. (**B**) Single-frame snapshot of low-force DNA molecule in molecular dynamics simulations. DNA tether end-to-end length of 5.5 μm (i.e., 35% contour length and a force of 0.07 pN). Highlighted regions (a) and (b) show the in cis and in trans recruitment events, respectively. Color coding is same as in (A). (**C**) Simulated kymographs representing distributions of ParB positions during the simulation. Top: Kymograph for DNA tether at 0.35 pN. Bottom: Same at *F* = 0.07 pN. Black arrow indicates an in trans recruitment event. (**D**) Probability density of ParB position from molecular dynamics simulations at different tether lengths (blue, 11 μm; red, 5.5 μm) averaged over *n* = 64 simulations. Dashed line indicates the position of dCas9 roadblock. (**E**) Fraction of simulated DNA tethers that exhibit ParB dimers passing the dCas9 roadblock at different cis/trans ratios. The theoretical bypassing rate (integral of the polymer looping probability; see fig. S8E) is shown in blue. Error bars represent SD across independent simulations (*n* = 128).

Using coarse-grained molecular dynamics simulations, we modeled the passage of DNA-bound roadblocks by in trans binding and found a similar strong force dependence of the passing fraction of roadblocks. We simulated DNA tethers containing a blocking particle that strictly prohibited ParB diffusion through it (Fig. 3A), whereas, concurrently, ParB could exhibit in cis or in trans recruitment with an inbuilt rate (see Fig. 3B and Materials and Methods). While, in the absence of a roadblock, both in cis and in trans recruitment increased the lateral spreading of ParB on the DNA (fig. S8A), the presence of a roadblock allowed ParB to spread beyond it only through in trans recruitment (Fig. 3, C and D, and fig. S8, B to D). Upon quantifying the passing fractions at different DNA end-to-end lengths and in cis/in trans recruitment ratios, we obtained the closest comparison with the experimental data at an in cis/in trans ratio of ~0.1 (Figs. 2G and 3E). Hence, the best agreement with the experimental data was obtained when in trans recruitment was assumed to lead to more successful events compared to in cis recruitment (Fig. 2G). Overall, the modeling at low forces verified the notion that contacts between regions on opposing sides of the roadblock occur sufficiently often to allow for ParB to overcome roadblocks via ParB-ParB recruitment.

### CTP hydrolysis in ParB-ParB recruitment

We found CTP hydrolysis to be crucial for the occurrence of ParB-ParB recruitment (Fig. 1H, fig. S9, and movie S2). After repeating the experiments at low CTP concentration of 10 μM, close to the dissociation constant (*K*_D_) of ParB-CTP (*19*), we observed a lower recruitment rate per loaded ParB dimer (fig. S9A), indicating that an exchange of CTP molecules may occur during the ParB-ParB recruitment process or that the recruited ParB needs to have CTP bound to be effectively recruited. To examine the role of CTP further, we replaced CTP by the poorly hydrolysable CTPγS (fig. S9B), which allows for the initial loading (clamp closing) but reduces the opening of the ParB clamp upon hydrolysis (fig. S9, C to F) (*4*, *7*, *19*, *25*). This resulted in a low and length-independent passing fraction, indicating an essential role of CTP hydrolysis in the in trans ParB-ParB recruitment (fig. S9, G to I). As CTP hydrolysis is tightly connected with ParB clamp reopening and dissociation from the DNA, it is likely that the N-terminal domain of ParB plays a role in the recruitment process. Recent work on ParB bridging interactions showed that a ParB^R80A^ mutant, deficient in CTP binding, does not efficiently form ParB-ParB bridges (*4*, *10*, *35*). As we hypothesize that ParB-ParB recruitment involves a transient bridge between the dimer proteins, the absence of open forms of ParB could explain the lack of recruitment with CTPγS in our experiments, because CTPγS binding keeps ParB proteins in a closed-clamp configuration. Furthermore, ParB mutants defective in CTP hydrolysis previously showed an altered distribution around *parS* in *B. subtilis*, with a reduced spreading against the orientation of highly expressed ribosomal RNA (rRNA) operons, genes encoding for ribosomal proteins, and tRNA operons, as well as an increased spreading in the opposite direction (*19*). We suggest that this distribution for the mutant ParB arises from 1D diffusion only (equivalent to the clamping and spreading model), while the spreading of the wild-type protein additionally relies on ParB-ParB recruitment that can overcome head-on encounters with protein machineries such as RNA polymerases or replication forks. Similar observations were made for ParB in *Caulobacter crescentus* (*25*), whereas by contrast, *Myxococcus* ParB mutants showed extensive spreading in both directions (*32*), possibly by unhindered 1D diffusion due to the differences in life cycles [i.e., lower gene transcription and replication rates; see (*36*, *37*)].

## DISCUSSION

### Model of ParB spreading in presence of DNA-bound roadblocks

**Fig. 4.**
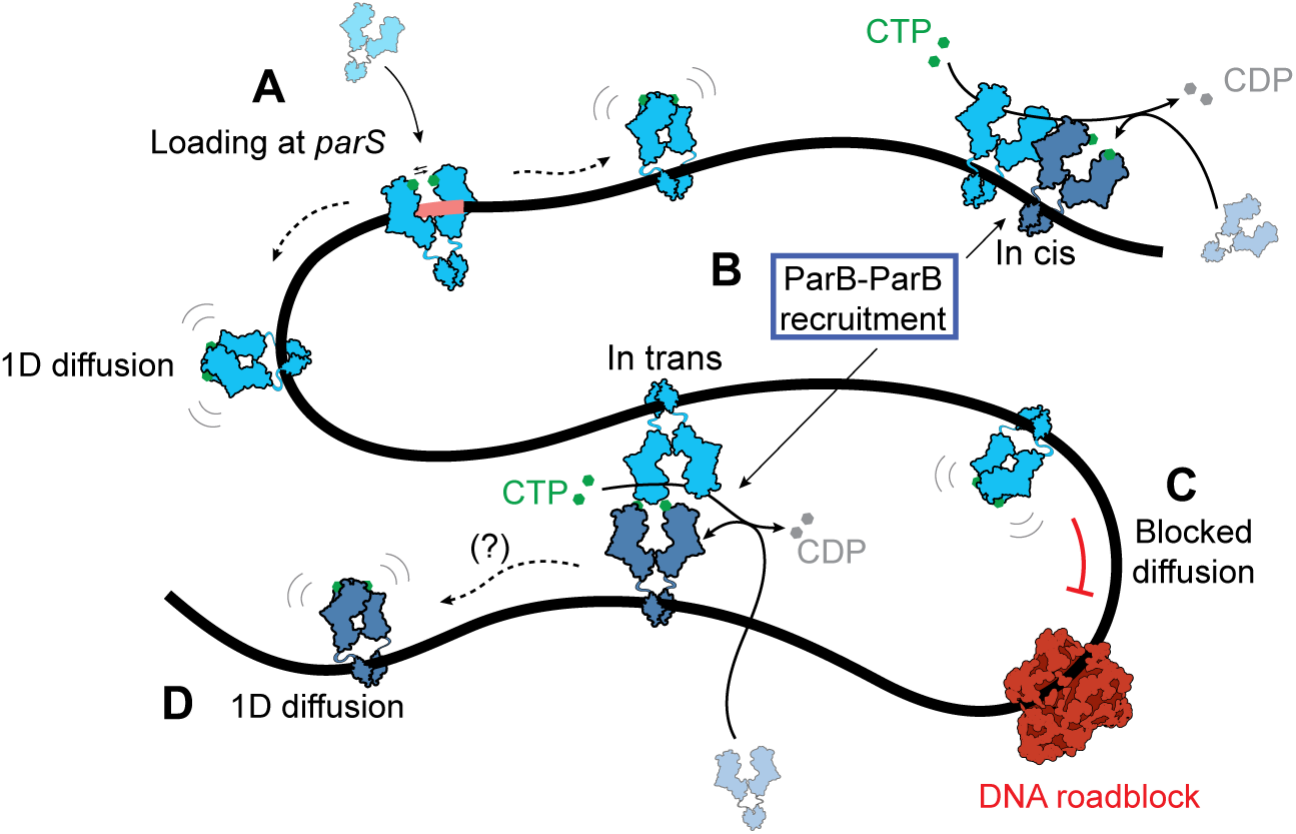
Model of ParB spreading and recruitment. Cartoon of the various ParB modalities on DNA: (**A**) ParB dimer loads at the *parS* site and dimerizes upon closing its N termini, losing its affinity to the *parS* site and diffusing away as a closed clamp. Subsequently, (**B**) ParB hydrolyzes CTP and enters an intermediary state where it can bind and recruit another ParB dimer to a nearby DNA, which can occur in cis or in trans. While 1D diffusion is blocked by DNA roadblocks (**C**), ParB dimers that are recruited in trans across the roadblock can continue diffusion along the DNA (**D**), yielding an expanded spreading of ParB along DNA. Whether ParB reforms a closed clamp structure after ParB-ParB recruitment (D) or not remains unknown.

On the basis of our findings, we propose a model for ParB spreading (Fig. 4) that expands the well-known clamping and sliding model. First, ParB loads onto the *parS* site and spreads away by 1D diffusion (Fig. 4A), until it hydrolyzes the bound CTP. In our model, however, unlike commonly assumed, the hydrolysis does not necessarily imply the immediate dissociation of ParB from the DNA. Instead, we hypothesize that the ParB dimer may briefly reside in an intermediary state after hydrolyzing CTP, where one of the monomers is not fully engaged in N-terminal dimerization (*19*). Given the abundant CTP in the surrounding buffer, the cytidine 5′-diphosphate can now exchange to a new CTP molecule, which leaves the CTP-bound free N-terminal part of the protein with two possibilities: (i) Either reconnect with the N terminus on the adjacent monomer of the ParB to reclose the clamp and continue diffusion (Fig. 4B) (*19*) or (ii) connect to the N terminus of a different ParB dimer that is nearby in the solution. The second scenario can lead to recruitment of a second ParB dimer to the nearby DNA, which can occur either in cis or in trans (Fig. 4C). In trans recruitment, in particular, would involve a transient loop formation between two distal segments on the DNA molecule. It has been previously proposed that ParB-like proteins can spread via a similar in trans mechanism (*38*), whereas previous work on ParB indicated that long loops may underlie ParB-DNA condensation (*39*), particularly at very low forces exerted in magnetic tweezer experiments (*7*, *31*, *33*) or DNA curtains (*10*, *13*). In our case, a loop would form transiently, and the ParB-ParB dimer-dimer connection would break following the recruitment event. In this way, in trans recruitment can result in DNA roadblock passing and free diffusion on the other side. The precise molecular mechanism of how the recruited ParB releases the dimer-dimer connection and whether it readopts a clamped conformation remain unknown (Fig. 4, C and D).

Overall, in trans ParB-ParB recruitment is consistent with the extensive spreading of ParB along genomic DNA that contains many roadblock proteins. Furthermore, it offers an alternative mechanism to the “stochastic binding” that was proposed previously (*28*) and expands on the combination of 1D and 3D interactions between ParB molecules in former models (*39*) to include the role of CTP and explain the presence of ParB at large distances from *parS* sites in the in vivo ChIP-seq data. We speculate that ideal hotspots for ParB in trans recruitment events may additionally be found in regions that are brought into close proximity of a *parS* site by the action of an Structural Maintenance of Chromosomes (SMC) complex (*40*, *41*). Recent reports on Hi-C maps detected in strains that were constructed for testing SMC collisions in vivo showed the coalignment of DNA flanking a *parS*-free region, which is characteristically only found at *parS* sites. Physical contact of DNA with a genomically distant *parS*-containing region may lead to in trans ParB-ParB recruitment and create *parS*-free ParB-DNA structures suitable for further off-site SMC recruitment (*42*). In line with our model, when the ParB-*parS* system was used for spot labeling in yeast or human cells, it would still spread (up to 3 kb) and form a bright fluorescent locus, despite the ubiquitous abundance of histones on the eukaryotic DNA molecule (*43*, *44*). This could be facilitated via recruitment of new ParB proteins to nearby DNA, overcoming the nucleosomes bound in between.

Our findings uncover a new pathway for ParB proteins to cooperatively cover large distances on DNA that is loaded with DNA binding proteins. It involves the combination of lateral 1D diffusion along the DNA and a new type of CTP hydrolysis–dependent ParB loading (ParB-ParB recruitment), which can occur irrespective of the *parS* site. Both our experimental and simulation data showed that in trans ParB-ParB recruitment can account for overcoming DNA-bound roadblocks at low forces where DNA forms a fluctuating polymer blob that facilitates frequent DNA-DNA encounters. As both the ParB concentration and the DNA-contact frequency are higher in the tightly packed genome within a bacterial cell compared to our single-molecule experiments, we expect ParB-ParB recruitment to be a common mechanism in vivo, where it may facilitate the collective spreading distance of ParB proteins on the protein-bound DNA.

## MATERIALS AND METHODS

### ParB purification and fluorescent labeling

We prepared *B. subtilis* ParB expression constructs using pET-28a–derived plasmids through Golden gate cloning. We expressed untagged recombinant proteins in *Escherichia coli* BL21-Gold (DE3) for 24 hours in ZYM-5052 autoinduction medium at 24°C. Purification of ParB^K252A, K255A, K259A^ (ParB^KKK^) and ParB^L5C^ variants was performed as described before (*19*). Briefly, we pelleted the cells by centrifugation and subjected them to lysis by sonication in buffer A [1 mM EDTA (pH 8), 500 mM NaCl, 50 mM tris-HCl (pH 7.5), 5 mM β-mercaptoethanol, 5% (v/v) glycerol, and protease inhibitor cocktail (Sigma-Aldrich)]. We then added ammonium sulfate to the supernatant to 40% (w/v) saturation and kept stirring at 4°C for 30 min. We centrifuged the sample and collected the supernatant and subsequently added ammonium sulfate to 50% (w/v) saturation and kept stirring at 4°C for 30 min. We collected the pellet (containing ParB) and dissolved it in buffer B [50 mM tris-HCl (pH 7.5), 1 mM EDTA (pH 8), and 2 mM β-mercaptoethanol]. The sample was also diluted with buffer B to achieve a conductivity of 18 mS/cm before loading onto a Heparin column (GE Healthcare). We used a linear gradient of buffer B containing 1 M NaCl to elute the protein. After collecting the peak fractions, we diluted them in buffer B to a conductivity of 18 mS/cm and loaded onto HiTrap SP columns (GE Healthcare). For elution, we used a linear gradient of buffer B containing 1 M NaCl. Collected peak fractions were loaded directly onto a Superdex 200-16/600 pg column (GE Healthcare) pre-equilibrated in 300 mM NaCl, 50 mM tris-HCl (pH 7.5), and 1 mM tris(2-carboxyethyl)phosphine (TCEP). For fluorescent labeling, we incubated purified ParB^L5C^ variant with either tetramethylrhodamine (TMR)-maleimide or Alexa Fluor 647-C_2_-malemeide at a 1:1.2 or 1:10 (protein:dye) molar ratio, respectively. We incubated the mixture for 15 min on ice, centrifuged for 10 min, then eluted from a spin desalting column (Zeba), and flash-frozen in liquid nitrogen. We estimated the fluorophore labeling efficiency at 74% for ParB^alexa647^ and 69% for ParB^R^ (resulting in 93 and 90% dimers labeled, respectively) by an inbuilt function on NanoDrop using extinction coefficients of ε = 270,000 cm^−1^ M^−1^ for Alexa Fluor 647 and ε = 60,000 cm^−1^ M^−1^ for TMR.

### ParB CTP hydrolysis assays

We test for the activity of the labeled ParB^L5C^ and ParB^KKK^ mutant by their ability to hydrolyze CTP in the presence of DNA*_parS_*. For this reason, we measured the hydrolysis rate by malachite green colorimetric detection. Briefly, we prepared a mixture of 2× [CTP] and 2× 40–base pair (bp) DNA*_parS_* concentration in reaction buffer [150 mM NaCl, 50 mM tris-HCl (pH 7.5), and 5 mM MgCl_2_] and placed it on ice. We added an equal volume of 2× solution, containing the purified protein to the 2× CTP/DNA mixture, and left to incubate at 25°C for 1 hour in a polymerase chain reaction (PCR) machine. In parallel, we prepared phosphate blanks. The samples were then diluted fourfold with deionized water, subsequently mixed with 20 μl of working reagent (Sigma-Aldrich), and transferred to a flat bottom 96-well plate. The plate was left to incubate at 25°C for 30 min, and we measured the absorbance at a wavelength of 620 nm. We used the absorbance values from the phosphate standard samples to plot an optical density of 620 nm versus phosphate concentration standard curve. Using the standard curve, we converted raw values to rate values and calculate the absolute rates by normalizing for protein concentration.

### Biolayer interferometry

Measurements were performed in a buffer containing 150 mM NaCl, 50 mM tris-HCl (pH 7.5), and 5 mM MgCl_2_ on BLItz machine (FortéBio Sartorius), similar to described previously in Antar *et al*. (*19*). Final protein concentrations in the association step (fig. S2I, c) were fixed at 1 μM for both ParB and the ParB^KKK^ mutants. All measurements were analyzed on the BLItz analysis software.

### Construction and purification of 42 kb DNA*_parS_* construct

For the construction of a long linear DNA*_parS_*, we used a large 42-kb cosmid-i95 previously reported (*45*) and a synthetic construct containing the *parS* site (Integrated DNA Technologies; table S1, underlined sequence). First, we linearized the i95 cosmid using the PsiI-v2 restriction enzyme (New England Biolabs). Next, we dephosphorylated the remaining 5′-phosphate groups using calf-intestinal alkaline phosphatase for 10 min at 37°C, followed by heat inactivation for 20 min at 80°C (Quick CIP, New England Biolabs). We added the 5′-phospho group on the synthetic *parS* fragment by adding a T4 kinase for 30 min at 37°C and heat-inactivated for 20 min at 65°C in 1× PNK buffer supplemented with 1 mM adenosine 5′-triphosphate (ATP) (T4 polynucleotide kinase, New England Biolabs). Next, we ligated the two fragments together using a T4 DNA ligase in T4 ligase buffer (New England Biolabs), containing 1 mM ATP overnight at 16°C. The final cosmid construct was transformed into *E. coli* NEB 10-beta cells (New England Biolabs), and we verified the presence of insert by sequencing using JT138 and JT139 (table S1). To prepare a linear fragment adapted for flow cell experiments, we isolated cosmid-i95 via a Midiprep using a QIAfilter plasmid midi kit (QIAGEN). The cosmid-i95 was then digested for 2 hours at 37°C and heat-inactivated for 20 min at 80°C using AjuI restriction enzyme (Thermo Fisher Scientific). Linear DNA constructs for three-color experiments with roadblocks were constructed in the same way using the SpeI-HF restriction enzyme (New England Biolabs). Next, we constructed the 5′-biotin handles by a PCR from a pBluescript SK+ (StrataGene) using 5′-biotin primers JT337 and JT338 (table S1) to get a final 1245-bp fragment. The PCR fragment was digested using the same procedure described for cosmid-i95, resulting in ~600-bp 5′-biotin handles. Last, we mixed the digested cosmid-i95 and handles in a 1:10 molar ratio and ligated them together using T4 DNA ligase in T4 ligase buffer (New England Biolabs) at 16°C overnight, which was subsequently heat-inactivated for 25 min at 65°C. We cleaned up the resulting linear (42 + 1.2 kb) DNA*_parS_* construct from the access handles using an ÄKTA Pure (Cytiva), with a homemade gel filtration column containing 46 ml of Sephacryl S-1000 SF gel filtration media, run with TE + 150 mM NaCl buffer at 0.2 ml/min. We stored the collected fractions as aliquots after snap-freezing them by submerging them in liquid nitrogen.

### Preparation and binding dCas9 roadblock to DNA*_parS_*

To form the dCas9-Alexa549 roadblock complex, we initially prepared a tr-crRNA duplex by mixing universal 67-nucleotide oligomer tracerRNA (trRNA) and a custom-designed crRNA (table S1) in a duplex buffer (Integrated DNA Technologies) to a final concentration of 10 μM each. The mixture was incubated at 95°C for 5 min and then slowly cooled to 4°C by decreasing the temperature for 5°C every 5 min over the course of 1.5 hours. We next incubated the tr-crRNA solution with the dCas9 in the “binding buffer” [2 μM tr-crRNA complex, 1 μM dCas9-SNAP (New England Biolabs), and 1× NEB3.1 buffer] for 10 min at 37°C before placing it on ice. Following the tr-crRNA-dCas9 complex formation, we bound it to the DNA*_parS_* by incubating the DNA*_parS_*:tr-crRNA-dCas9 in molar ratio of 1:50 for 60 min at 37°C. We labeled the dCas9-SNAP by adding Alexa546-O^6^-benzylguanine (BG) (or Alexa Fluor 647-BG for photobleaching estimation) to a final concentration of 1 μM for 30 min at room temperature before flowing it into the flow cell.

### Single-molecule visualization assay

The surface of imaging coverslips was prepared as previously described (*46*), with the addition of surfaces being PEGylated 5× for 24 hours. For immobilization of 42-kb DNA*_parS_*, we introduced 50 μl of ~1 pM 5′-biotinylated DNA*_parS_* molecules at a flow rate of 3 to 14 μl/min, depending on the desired end-to-end length in the experiment, in T20 buffer [40 mM tris-HCl (pH 8.0), 20 mM NaCl, and 25 nM SYTOX Green (SxG; Thermo Fisher Scientific)]. Immediately after the flow, we further flowed 100 μl of the wash buffer [40 mM tris-HCl (pH 8.0), 20 mM NaCl, 65 mM KCl, and 25 nM SxG] at the same flow rate to ensure stretching and tethering of the other end of the DNA to the surface. By adjusting the flow, we obtained a stretch of around 25 to 80% of the contour length of DNA. Next, we flowed in the imaging buffer [40 mM tris-HCl, 2 mM trolox, 1 mM TCEP, 10 nM catalase, 18.75 nM glucose oxidase, 30 mM glucose, 2.5 mM MgCl_2_, 65 mM KCl, bovine serum albumin (0.25 μg/ml), 1 mM CTP, and 25 nM SxG] without ParB protein at the same flow rate to maintain identical conditions before and after protein addition. Experiments were performed under the same conditions with the exception of replacing 1 mM CTP with 10 μM CTP or 1 mM CTPγS where mentioned. Real-time observation of ParB diffusion was carried out by introducing ParB (0.05 to 1 nM) in the imaging buffer. We used a homebuilt objective total internal reflection fluorescence (TIRF) microscope to achieve fluorescence imaging. We used alternating excitations of 488 and 646 nm, as well as 561-nm lasers in HiLo microscopy mode, to image SxG-stained DNA and Alexa Fluor 647- or TMR-labeled ParB. When imaging Alexa488-ParB, we used continuous 488-nm excitation in the absence of SxG. All images were acquired with a Prime BSI scientific CMOS (complementary metal-oxide semiconductor) camera at an exposure time of 100 ms for dual-color experiments and 60 ms for three-color experiments, with a 60× oil immersion and 1.49 numerical aperture CFI APO TIRF (Nikon).

### Crossed DNA*_parS_*-DNA_X_ assay

The surface was prepared, and binding of the DNA*_parS_* was done the same as described above. The flow cell was then blocked at the primary inlet (fig. S7) and resumed from a secondary inlet to establish a cross flow that was oriented under a substantial angle with the flow that stretched DNA*_parS_* on the surface. In this second flush, DNA that contained no *parS* site was stretched onto the surface until sufficient number of crossed DNA*_parS_*-DNA_X_ events was observed. We performed the remainder of the experiment identically to the previously described protocol with a fixed concentration of 500 pM ParB.

### Image processing

The areas with single DNA molecules were cropped from the raw image sequences and analyzed separately with a custom-written interactive Python software. The images were smoothened using a median filter with a window size of 10 pixels, and the background was subtracted with the “white_tophat” operation provided in the SciPy Python module. The contrast of the images was further adjusted manually for visualization only (i.e., Fig. 1F, bottom). The ends of a DNA were manually marked. Total fluorescence intensity of 11 pixels across the axis of the DNA was obtained for each time point and was stacked to get a kymograph (i.e., Fig. 1B). The same DNA axis was chosen to obtain kymograph of the ParB fluorescence channel.

### Data analysis of single-molecule imaging traces

We resolved the initial loading positions of ParB by calculating the mean pixel position over the first full second of the ParB traces from the kymograph and then determining that value relative to the DNA ends, i.e.

$$\text{Relative loading position}=\frac{\left(x_{\text{end}}-x_{\text{load}\;\text{avg}.}\right)}{\left(x_{\text{end}}-x_{\text{start}}\right)}$$ (1)

All loading positions obtained like this were pulled together and represented in a histogram using a custom-written Python script (Fig. 1D and figs. S2, B and H, and S9). We determined the residence time of ParB molecules by measuring the length of traces of single ParB proteins by their fluorescence (Fig. 1E and figs. S3, B to D; S4, C and D; and S9F). We fitted the histogram with a two- or three-parameter model and computed the Bayesian information criterion (BIC; fig. S3, B and C) for each fit to determine the optimal model underlying the data. The mean was obtained via bootstrapping of all data (*n*_iterations_ = 5000; Fig. 1E). The SEM was computed using the following formula

$$\text{SEM}=\frac{\text{SD}}{\sqrt{\text{Sample size}}}$$ (2)

Similarly, we obtained the ParB residence time before and after recruitment (fig. S4, C and E).

We obtained the *parS* and non-*parS* arm intensities in dCas9 roadblock experiments (Fig. 2, C and E) by selecting the corresponding regions in the kymograph before (~100 frames) and after (~2000 frames) the observable binding of the first ParB molecule to the DNA. Pixel values before the initial binding, representing noise, were averaged over the 100 frames and subtracted from each frame in the region after the initial molecule bound. The roadblock position was determined as the maximum value over the time-averaged kymograph in the 561-nm channel after applying the Savitzky-Golay filter implemented from the SciPy Python module, with a window length of 11 frames and an order of 1. ParB signal intensity was obtained by applying a median filter of the kymograph from a 647-nm channel with a kernel size of 21 frames to account for the signal noise and the noise from the wiggling of the DNA molecule. We discarded the intensity data from a window of five pixels above and below the dCas9 position to account for the “leaking” fluorescence due to fixed pixel position of dCas9 (at the peak of Savitzky-Golay curve) and simultaneous wiggling DNA and ParB signal with it. The raw data were plotted on the same graph as the median filter curves in the background.

To determine the fluorescence intensity of ParB proteins before, during, and after recruitment events, single discernable ParB proteins were tracked on kymographs as described above. We then integrated the fluorescence from the three surrounding pixels of the track position for 20 frames before, during, and after the recruitment event. For visualization of example recruitment events (fig. S4B), we temporally averaged 20 frames of kymographs to obtain a profile across the DNA length and identified one or two peaks (before/during and after recruitment, respectively) by calling a Gaussian mixture model from the Python sklearn module with the respective number of components.

### Modeling the distribution of residence times

To model the distribution of residence times of ParB on DNA in saturating concentration of CTP of 1 mM, we consider the pathway depicted in fig. S3A. Starting with the saturated ParB dimer on the DNA, we assume that the CTP hydrolysis in the two monomers occurs independently with the rate constant *k*_CTP_. Once both CTP molecules are hydrolyzed, ParB is released from the DNA with the rate *k*_off_. To model the independent hydrolysis of the two CTP molecules, we assume that the CTP hydrolysis follows single exponential kinetics

$$P(t)=k_{\text{CTP}}e^{-k_{\text{CTP}}t}$$ (3)

The time until both CTP molecules are hydrolyzed (*t*_CTP_) then corresponds to the maximum of two exponential random variables with the rate *k*_CTP_. The distribution of *t*_CTP_ is given by the probability that either one of the two CTP molecules is hydrolyzed at a time point *t*_CTP_, multiplied by the probability that the other CTP molecule was already hydrolyzed at an earlier time point, i.e.

$$P(t_{\text{CTP}})=2P(t=t_{\text{CTP}})\;P(t\leq t_{\text{CTP}})$$ (4)

where the factor 2 accounts for the fact that either of the ParB monomers might hydrolyze at first, from which we obtain

$$P(t_{\text{CTP}})=2k_{\text{CTP}}e^{-k_{\text{CTP}}t}(1-e^{-k_{\text{CTP}}t})$$ (5)

We assume that the release of the ParB dimer from the DNA after hydrolysis of both CTP molecules occurs with a constant rate *k*_off_

$$P(t_{\text{off}})=k_{\text{off}}e^{-k_{\text{off}}t}$$ (6)

The total dwell time of ParB on the DNA is then given by *T* = *t*_CTP_ + *t*_off_. The distribution of the dwell time *T* is obtained from the convolution of *P*(*t*_CTP_) and *P*(*t*_off_)

$$P(T)=\int_{0}^{T}P(t_{\text{CTP}}=t)\;P(t_{\text{off}}=T-t)\text{dt}$$ (7)

which evaluates to

$$\begin{array}{c} P(T)=2k_{\text{CTP}}k_{\text{off}}e-k_{\text{off}}T\\ \left[{\left(k_{\text{CTP}}-k_{\text{off}}\right)}^{-1}(1-e^{-(k_{\text{CTP}}-k_{\text{off}})T})-{\left(2k_{\text{CTP}}-k_{\text{off}}\right)}^{-1}\;(1-e^{-{\left(2k_{\text{CTP}}-k_{\text{off}}\right)}^{T}})\right] \end{array}$$ (8)

To account for the photobleaching of the fluorophore, we consider the apparent dwell time *T*′ = min (*T*,*t*_bl_) as the minimum of the dwell time *T* and the exponentially distributed bleaching time *t*_bl_

$$P(t_{b1})=k_{b1}e^{-k_{b1}t_{b1}}$$ (9)

where *k*_bl_ is the bleaching rate. The distribution of the apparent dwell time is then obtained as

$$P(T\prime =\text{min}(T,t_{b1}))=P(T=T\prime )P(t_{b1}>T\prime )+P(t_{b1}=T\prime )(T>T\prime )$$ (10)

Here, *P*(*T* = *T*′)*P*(*t*_bl_ > *T*′) is the probability that ParB dissociates from the DNA before photobleaching occurs, and *P*(*t*_bl_ = *T*′)*P*(*T* > *T*′) is the probability that photobleaching occurs during the dwell time of ParB on the DNA, with

$$P(t_{b1}>T\prime )=1-P(t_{b1}\leq T\prime )=1-\int_{0}^{T}P(t_{b1}=t)\text{dt}=e^{-k_{b1}T\prime }$$ (11)

and accordingly

$$\begin{array}{c} P(T>T\prime )=1-\frac{2k_{\text{CTP}}k_{\text{off}}}{\left(2k_{\text{CTP}}-k_{\text{off}})\;(K_{\text{CTP}}-K_{\text{off}}\right)}\\ \left[\frac{k_{\text{CTP}}}{k_{\text{off}}}(1-e^{-k_{\text{off}}T})-\frac{2k_{\text{CTP}}-k_{\text{off}}}{k_{\text{CTP}}}(1-e^{-k_{\text{CTP}}T})+\frac{k_{\text{CTP}}-k_{\text{off}}}{2k_{\text{CTP}}}(1-e^{-2k_{\text{CTP}}T})\right] \end{array}$$ (12)

The same procedure was applied account for bleaching in the case of immediate release of ParB from the DNA according to Eq. 5.

### Fitting of the dwell time distributions

To avoid artifacts related to the binning of the data, we applied a maximum likelihood approach to fitting the experimentally obtained apparent dwell times *t*′. The log likelihood of the data, given the model function as given by Eq. 10, is given by

$$\text{log}ℒ=\sum \text{log}P(T\prime ={t\prime }_{i})$$ (13)

Optimization of the log likelihood with respect to the rates of CTP hydrolysis (*k*_CTP_) and release (*k*_off_) was performed using the interior point algorithm as implemented in the fmincon function in MATLAB. The bleaching rate *k*_bl_ was fixed (*k*_bl_ = 1.75 × 10^−4^ s^−1^ for Alexa Fluor 647, extracted from our experimental data, for TMR this rate was fixed to *k*_bl_ = 0 s^−1^). Confidence intervals (68%) were determined from the Hessian at the solution. To compare different model functions with varying parameters (i.e., immediate versus delayed release from the DNA), we used the BIC defined as

BIC=νlogN−2logℒ (14)

where ν is the number of fit parameters (ν = 1 for immediate release and ν = 2 for delayed release) and *N* is the number of data points. The model with the lowest value for the BIC was preferred.

### CTP hydrolysis rates and ParB dissociation rates

We fitted the experimental residence time distributions for ParB^R^ and ParB^alexa647^ to the immediate release (Eq. 5) and delayed release (Eq. 8) models (table S2 and fig. S3, B and C). For both dyes, the delayed-release model was clearly preferred on the basis of the lower value for the BIC. The CTP hydrolysis rates for the delayed-release model were consistent between ParB^R^ (*k*_CTP_ = 0.022 ± 0.003 s^−1^) and ParB^alexa647^ (*k*_CTP_ = 0.025 ± 0.003 s^−1^) but faster compared to the bulk rates of ~0.007 s^−1^ that we measured (fig. S1F) and previous reports of ~0.01 s^−1^ (*4*). On the other hand, we obtained different dissociation rates *k*_off_ for ParB^R^ (0.025 ± 0.005 s^−1^) and ParB^alexa647^ (0.06 ± 0.02 s^−1^), corresponding to dwell times of the ParB dimer on DNA after CTP hydrolysis of 40 ± 8 s for ParB^R^ and 17 ± 5 s for ParB^alexa647^. While the CTPase activity remained unaffected by the dye labeling (fig. S1F), this suggests that the organic dye could promote the DNA binding affinity of the ParB dimer after clamp opening. Together, we estimate a dissociation rate of *k*_off_ = 0.04 ± 0.02 s^−1^ (dwell time, 25 ± 13 s).

### Accidental recruitment colocalization simulations

Because the recruitment of ParB molecules is visually indistinguishable from a colocalization of a newly recruited ParB dimer onto the location of an already bound ParB, we analyzed whether a random colocalization of ParB’s might give rise to the experimentally observed recruitment frequency. To this end, we simulated kymographs mimicking DNA being tethered at an end-to-end length, which was used for experiments. A molecule was allowed to bind at the *parS* site (at a relative DNA position of 0.5) and undergo 1D diffusion with an experimentally determined diffusion constant *D* = 0.066 μm^2^/s (fig. S2E) and an average residence time of 76 s (Fig. 1E), drawn from a normal distribution with σ = 50 *s*. The number of *parS*-loaded ParB’s and nonspecifically bound ParB’s per kymograph of 7500 frames (equivalent to 1500 s) was drawn from a normal distribution, with a mean of 4 and an SD of 1 molecule, in line with the experimentally observed value. Nonspecifically bound molecules were allowed to bind anywhere along the DNA with equal probability. Such scenarios were simulated a thousand times (fig. S4D shows an example), and colocalization of *parS*-loaded and nonspecifically bound ParB molecules was counted if the two molecules came closer than 300 nm from each other within three frames in time, accounting for our diffraction limit during experiments, below which the two molecules cannot be distinguished (we estimate that the point spread function of our microscope is roughly 300 nm wide).

### Modeling and molecular dynamics simulations

We modeled the DNA as a coarse-grained semiflexible polymer made of 1400 spherical beads of size σ = 5.5 nm = 16 bp. We placed the *parS* recruitment sequence in the middle of the chain. In addition, at two-thirds of the DNA chain, we placed a roadblock that stopped the diffusion of ParB, representing the dCas9 enzyme. The beads interact purely by excluded volume following the shifted and truncated Lennard-Jones (LJ) force field

$$U_{\text{LJ}}(r,\sigma )=\{\begin{array}{c} 4\varepsilon [{\left(\frac{\sigma }{r}\right)}^{12}-({\frac{\sigma }{r}}^{6}+\frac{1}{4})]\;\text{for}\;r\leq r_{c}\\ 0\;\text{otherwise} \end{array}$$ (15)

where *r* denotes the distance between any two beads and $r_{c}=2^{\frac{1}{6}}\sigma$ is the cutoff. We defined the bonds between two monomers along the DNA contour length by the finite extensible nonlinear elastic (FENE) potential, given by

$$U_{\text{FENE}}(r)=-0.5kR_{o}^{2}\text{log}(1-{\left(\frac{r}{R_{o}}\right)}^{2})\;\text{for}\;r\leq R_{o}$$ (16)

with *k* = 30ε/σ^2^ as the spring constant, ε is thermal energy, and *R*_o_ = 1.5σ is the maximum length of the bond. We introduced the persistence length of the DNA chain as a bending potential energy between three consecutive beads given by

$$U_{\text{bend}}(\theta )=k_{\theta }(1-\text{cosθ})$$ (17)

where θ is the angle between two consecutive bonds and *k*_θ_ = 10 *k*_B_*T* is the bending stiffness, corresponding to a persistence length of about 10 beads or ~55 nm. We modeled ParB by calling, within the LAMMPS engine, an external program that modifies the types of the beads along the DNA over time and thereby mimics the diffusion/recruitment of ParB. Specifically, at *t* = 0, we loaded a ParB protein onto the *parS* site and allowed to diffuse with a constant $D=0.05\frac{\mu m^{2}}{s}=0.05\;(\frac{182^{2}\sigma^{2}}{2.8\;10^{8}\text{dt}})=0.062\frac{\sigma^{2}}{10^{4}\text{dt}}$. This is done by updating the position of a loaded ParB protein either to the left or to the right with probability 0.125 every 10^4^ dt = 10^2^T_B_ timesteps (recall that mean squared displacement = 2 *Dt* in a 1D system, hence why the jump probability is twice the diffusion coefficient *D*). The diffusion cannot happen (the move is rejected) if the attempt brings a ParB protein either on top of another ParB or on top of dCas9.

On top of diffusion, we add a recruitment process at a rate of $10^{-6}\tau_{B}^{-1}$, i.e., on average, every 10^6^τ_B_ time steps (0.35 s), in which another ParB is recruited by a loaded ParB. When this happens, the recruitment can stochastically happen in cis (with probability *p*_c_) or in trans (with probability *p*_T_ = 1 − *p*_C_). If the former is selected, then one of the two adjacent beads is selected at random; and if unoccupied and not the dCas9 bead, then a new ParB is added onto the chain. Otherwise, if the latter “in trans” mechanism is selected, then we compute the list of 3D proximal neighbors that must be (i) within an Euclidean cutoff distance of 11 nm (roughly the size of a two ParB dimers) and (ii) farther than the second-nearest neighbor in 1D (i.e., the first- and second-nearest neighbors cannot be picked to avoid in trans events that are in practice in cis). The “theoretical” bypass probability is given by the integral of the looping probability (e.g., Yamakawa-Shimada *J* factor) (fig. S8E) as

$$P_{\text{bypass}}\propto \int_{2}^{\infty }P_{\text{loop}}(x)\mathrm{dx}$$ (18)

Once the list of 3D neighbors is compiled, we randomly pick one of these from the list (if not empty), load a new ParB protein, and resume the Langevin simulation.
