# Supplementary material for: ParB proteins can bypass DNA-bound roadblocks via dimer-dimer recruitment
Source: Sci Adv. 2022 Jun 29;8(26):eabn3299. doi: 10.1126/sciadv.abn3299 (PMC9242446; doi:10.1126/sciadv.abn3299)
Supplement: Supplementary file 1 — Figs. S1 to S9 Tables S1 and S2 [file sciadv.abn3299_sm.pdf]

Science Advances
AAAS

Supplementary Materials for

ParB proteins can bypass DNA-bound roadblocks via dimer-dimer recruitment

Miloš Tišma *et al.*

Corresponding author: Cees Dekker, c.dekker@tudelft.nl

Sci. Adv. **8**, eabn3299 (2022)
DOI: 10.1126/sciadv.abn3299

The PDF file includes:

Figs. S1 to S9
Tables S1 and S2
Legends for movies S1 to S4

Other Supplementary Material for this manuscript includes the following:

Movies S1 to S4

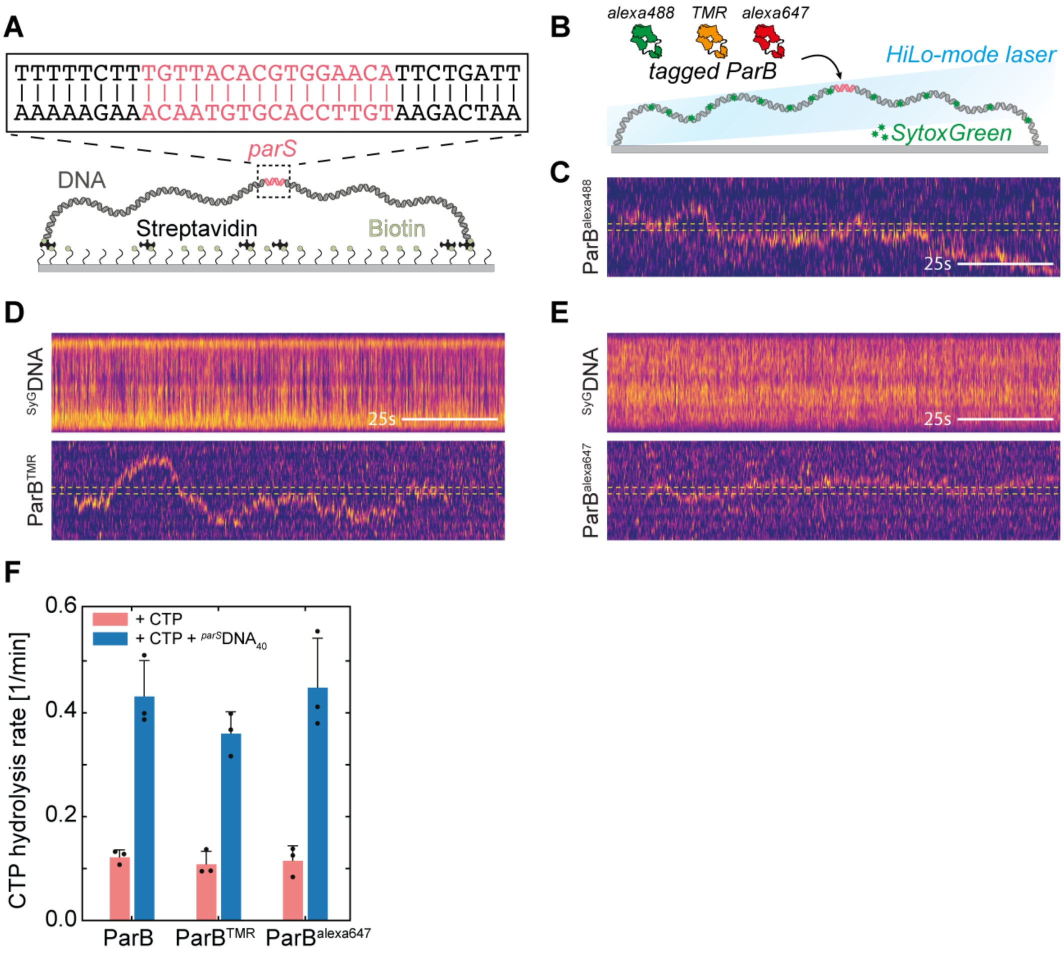

Figure S1. ParB exhibits one-dimensional diffusion irrespective of the fluorescent tag. A) Schematic representation of the DNA*parS* that is tethered at both its ends to a surface. Zoomed region shows *parS* sequence. **B)** Schematic representation of HiLo imaging setup using different fluorescent tags on ParB as well as SytoxGreen for DNA*parS* staining. **C)** Kymograph showing ParB diffusion using ParB^{alexa488}. Scale bar = 25s. yellow line - *parS* position (mirrored) **D)** and **E)** Kymographs for DNA*parS* stained with SytoxGreen (top) and ParB^{TMR} (bottom) or ParB^{alexa647} (bottom), respectively. Scale bar = 25s. yellow line - *parS* position (mirrored) **F)** CTP hydrolysis assay with Malachite Green for unlabelled, TMR- and Alexa647-labelled ParB proteins. The rates were normalized to the blank phosphate controls (see Methods). Error bars represent standard deviation from a triplicate (presented as black circles).

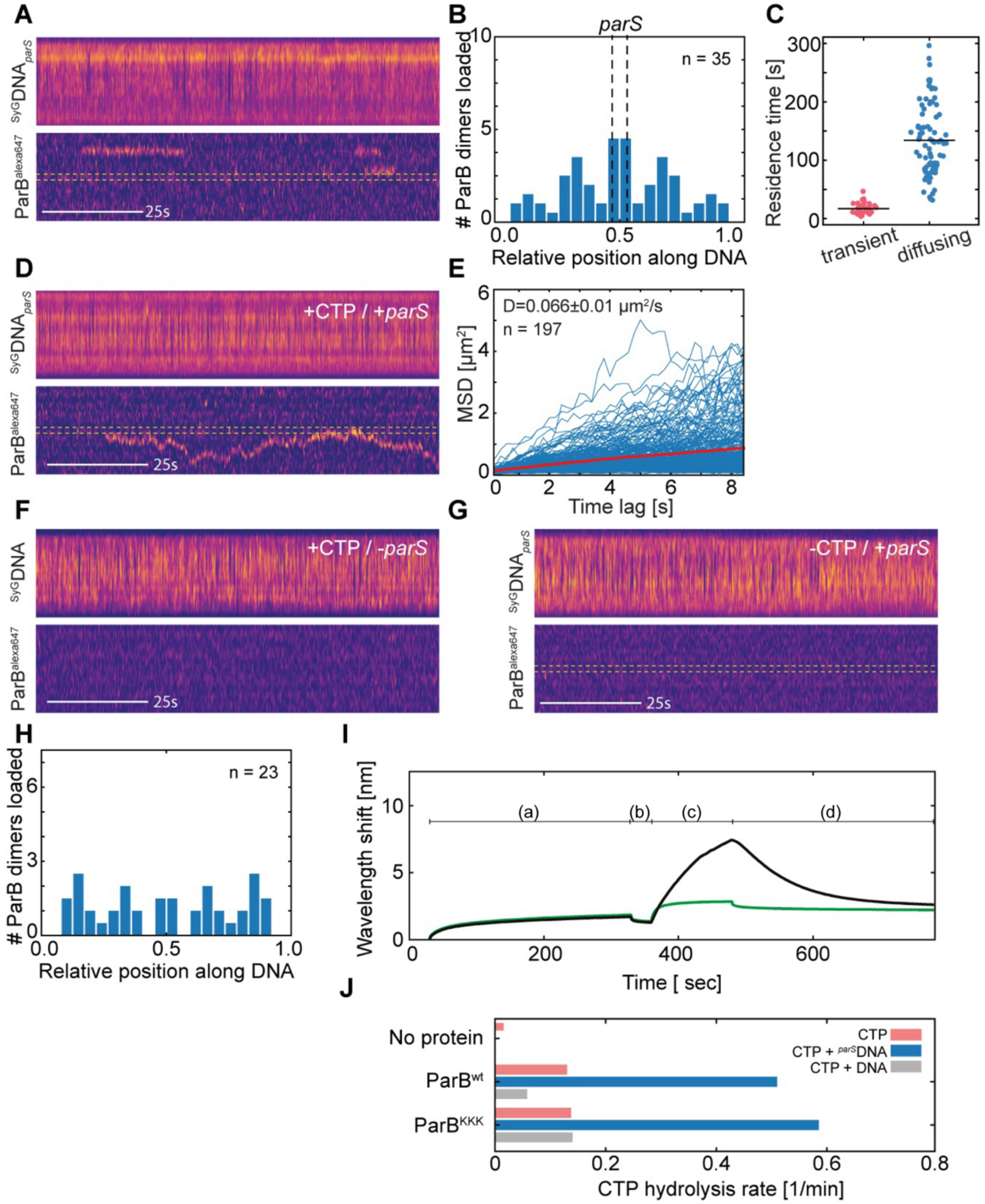

Figure S2. ParB spreading by diffusion occurs in the presence of *parS* sequence and CTP. A) Kymographs for DNA_{parS} stained with SytoxGreen (top) and $ParB^{alexa647}$ (bottom). Yellow dashed line indicates the *parS* site (mirrored). **B)** Mirrored histogram representing transient binding position of ParB dimer molecules relative to the DNA_{parS} ends. The events are double counted and hence display a symmetrical distribution as the head-to-tail orientation of the DNA is unknown. The position of *parS* site is represented by dashed lines. n=35. **C)** Residence times of transiently bound non-diffusing (pink) and *parS*-bound and diffusing ParB dimers (blue). **D)** Kymographs for DNA_{parS} stained with

SytoxGreen (top) and ParB-alexa647 (bottom) in the presence of both CTP and *parS* site. Yellow dashed line indicates the *parS* site (mirrored). **E)** Mean square displacement of the diffusing ParB molecules loaded at *parS* site. Red line represents a linear fit to the MSD curves. Apparent diffusion coefficient is on average D= 0.066±0.01 $\mu m^2/s$, n= 197. **F)** Kymographs for DNA_{parS} stained with SytoxGreen (top) and $ParB^{alexa647}$ (bottom) in the absence of *parS*-site. **G)** Kymographs for DNA_{parS} stained with SytoxGreen (top) and $ParB^{alexa647}$ (bottom) in the absence of CTP. Yellow dashed line indicates the *parS* site. **H)** Mirrored histogram for the binding position on non-specifically bound ParB dimers (+CTP/-*parS*) relative to the DNA_{parS} ends. The events are double counted and hence display a symmetrical distribution as the head-to-tail orientation of the DNA is unknown. **I)** Biolayer interference showing DNA_{parS} binding of ParB (black) and $ParB^{KKK}$ mutant (green). (a) Binding of biotin-immobilized 169bp DNA_{parS}; (b) buffer only; (c) protein association step as 1 µM protein and 1 mM CTP was added to buffer; and finally, during dissociation (d), an equivalent buffer lacking ParB protein and nucleotide was applied (see Methods and *Antar et al* (*1*)). **J)** CTP hydrolysis assay with Malachite Green for ParB and $ParB^{KKK}$ mutant.

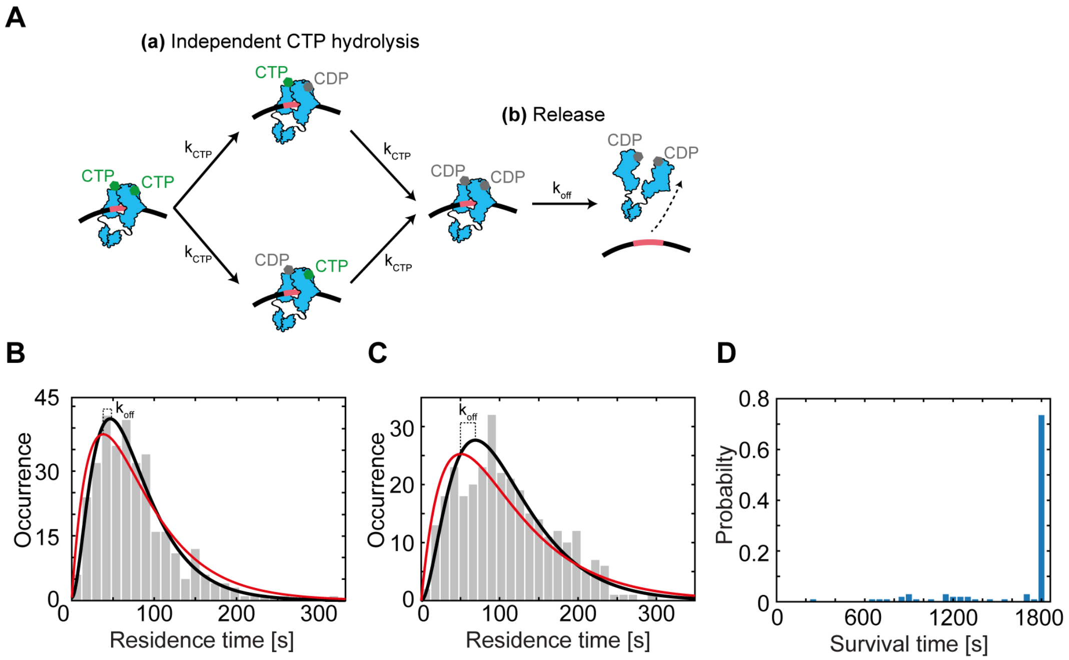

Figure S3. Model for the release of the ParB dimer from DNA after CTP hydrolysis. A) Following hydrolysis of both CTP molecules with equal rates k_{CTP}, the ParB dimer releases from the DNA with rate k_{off}. **B)** Fit to the experimental residence time distribution for $ParB^{alexa647}$ to the model in A) assuming immediate (red, BIC=3493) or delayed (black, BIC=3418) dissociation of ParB from the DNA after CTP hydrolysis. **C)** Same as in B) for $ParB^{TMR}$. For immediate release (red) the BIC is 3382, while for delayed release BIC = 3363. The results of the fits are given in Table S2. **D)** Survival rates of dCas9-alexa647 proteins undergone the same imaging procedure as ParB proteins. Bleaching rate was very low, $k_{bl} = 0.00018\ s^{-1}$.

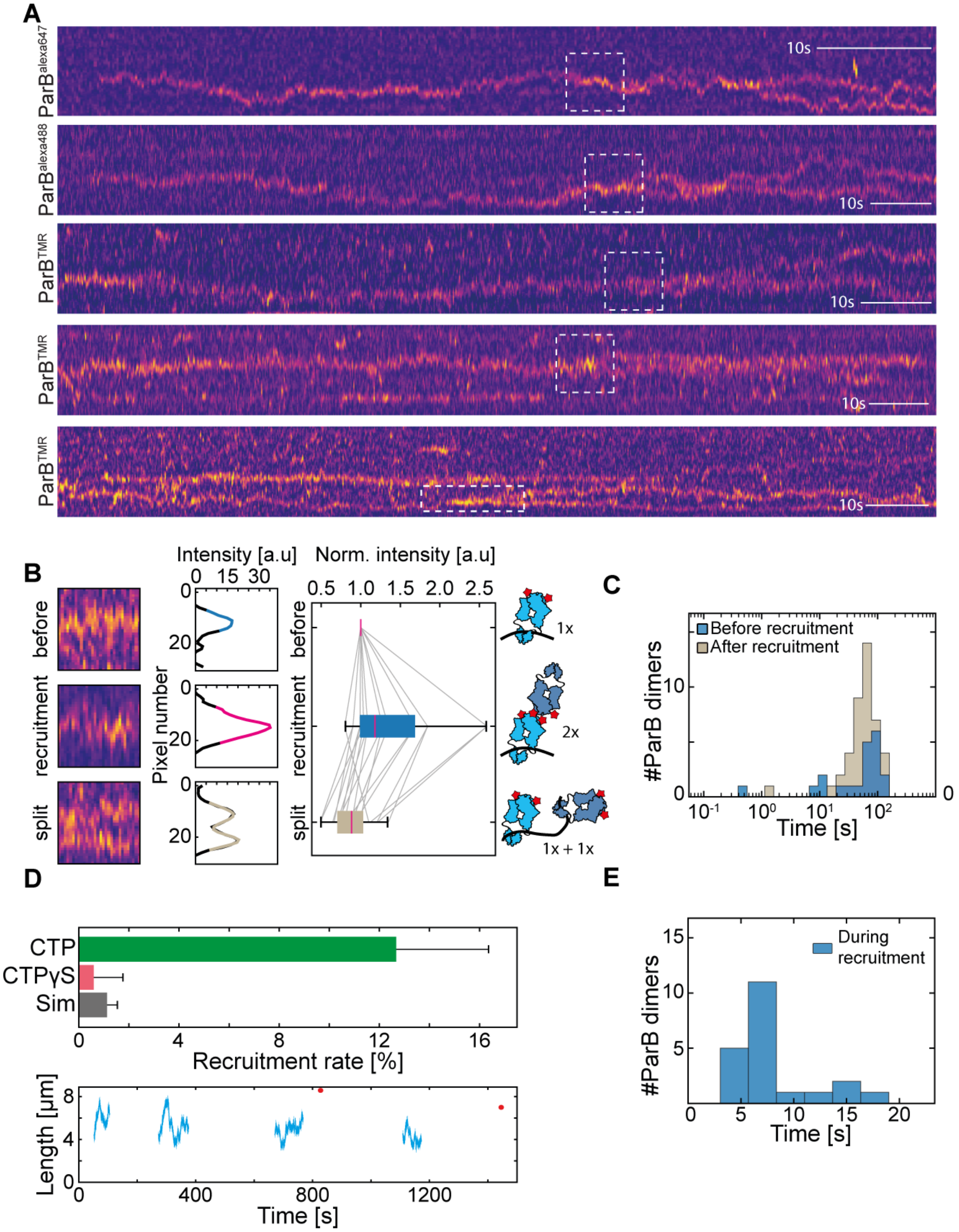

Figure S4. ParB-ParB recruitment is marked by short conjunct diffusion followed by a split and two individually diffusing ParB dimers. A) Kymographs for ParB-ParB recruitment using ParB proteins with different tags. Top-to-bottom: ParB-alexa647, ParB-alexa488, 3xParB-TMR. Scale bar = 10s. Dashed boxes represent ParB-ParB recruitment event. **B)** Left: exemplary parts of the kymographs "before recruitment", "during recruitment" and "split after recruitment" from traces such in panel A). Window size is 25px*25 frames. Middle: Mean intensity

projection from the windows on the left. Right: Relative intensity "during recruitment" and "split after recruitment", as normalized to the "before recruitment" of the same kymograph. n=25 before and n=50 after splitting. Cartoon representations on the right represent the ParB dimerization that explains the increased signals during recruitment. **C)** Residence time of single ParB dimers before (blue) and after (cream) recruitment events. **D)** Simulated traces with experimental parameters to estimate the "accidental colocalization" probability. Top – colocalization events occurred in presence of CTP, CTPγS and in our *in-silico* experiment. Bottom – exemplary trace of our colocalization simulations **E)** Time duration of the conjunct diffusion period during the recruitment event.

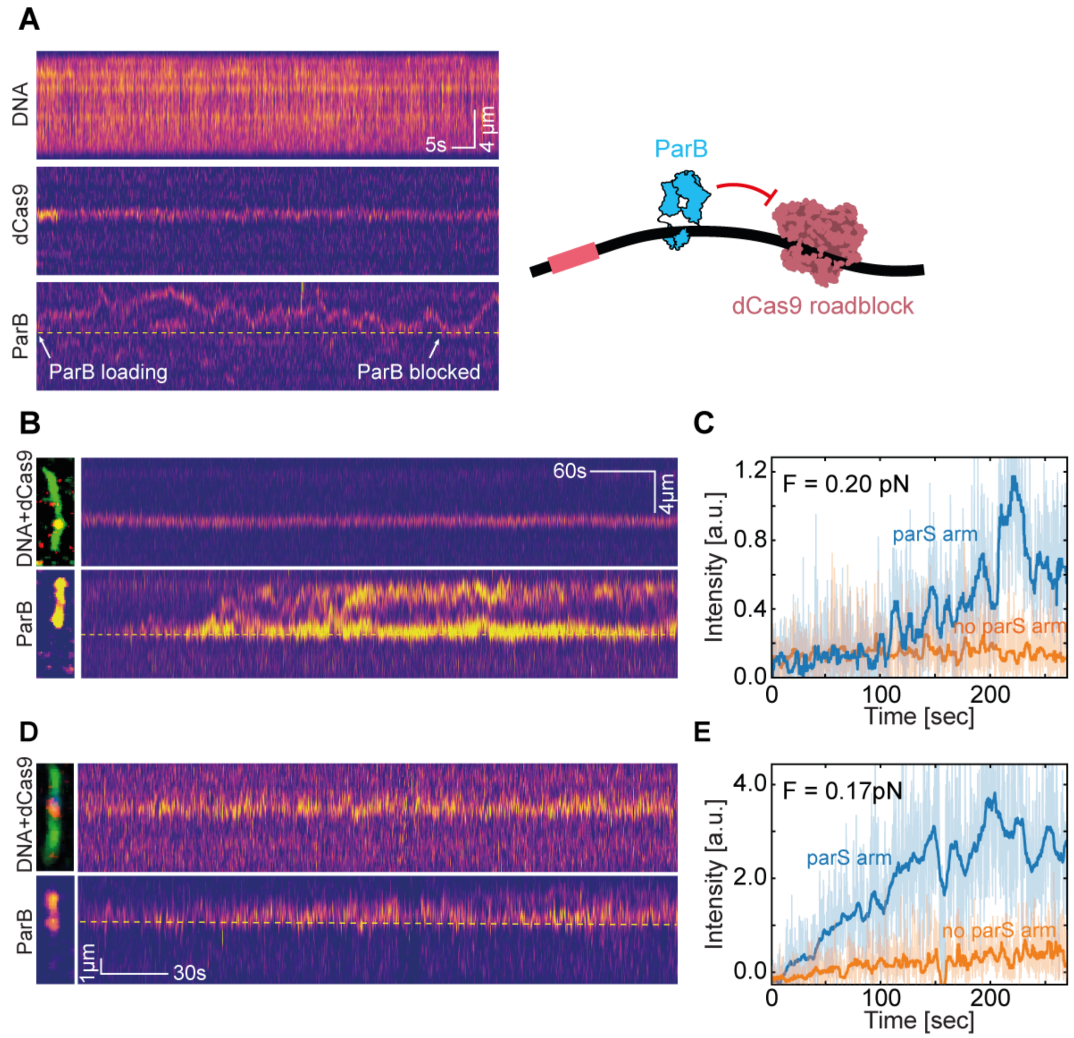

Figure S5. DNA-bound dCas9 roadblocks efficiently block diffusion of ParB dimers. A) Kymographs for DNA$_{parS}$ stained with SytoxGreen (top) and dCas^{alexa549} (middle) and ParB^{TMR} (bottom) at low concentration [0.1nM]. White arrows indicate the loading position (left) and the moment of dCas9 blocking ParB diffusion (right). Cartoon representation shown on the right. Scale bars: spatial 4µm, temporal 5s. **B)** Kymographs for dCas^{alexa549} (top) and ParB^{alexa647} (bottom) at 10x higher ParB concentration [1nM]. Left images are snapshots of dCas9^{alexa549} & ^{SytoxGreen}DNA$_{parS}$ overlay, and ParB^{alexa647} signal at the end of the trace. Yellow dashed line indicates the position of the dCas9^{alexa549}. Scale bars: spatial 4µm, temporal 60s. **C)** Quantification of the kymograph data of panel B, that displays the ParB signal in the top (blue, raw data – light blue) and bottom (orange, raw data – light orange) part of the DNA, i.e., above and below the Cas9 (dashed yellow line), respectively, at F = 0.20 pN. **D)** Same as panel B. **E)** Same as panel C**.**

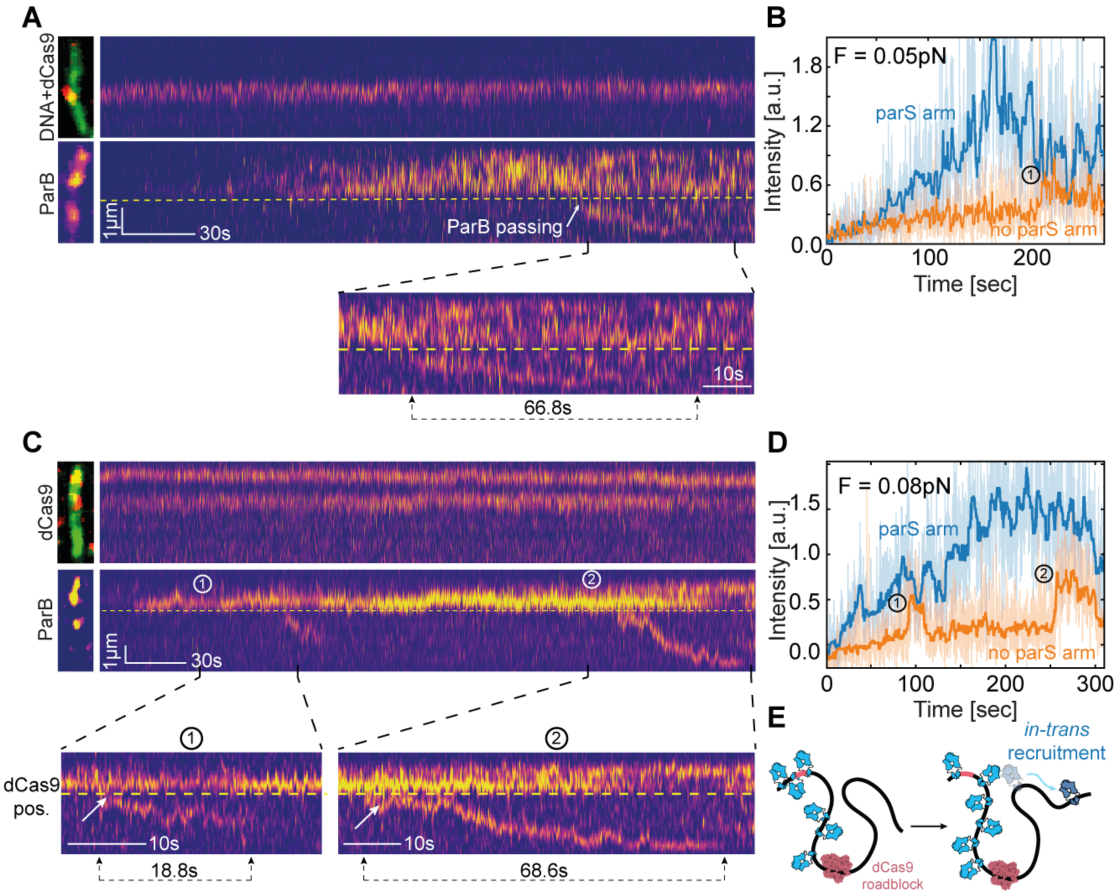

Figure S6. ParB-ParB in-trans recruitment allows for bypassing DNA-roadblocks at low forces. A) Kymograph for dCas9^{alexa549} (top), and ParB^{alexa647} (bottom) for DNA$_{parS}$ (same as Fig.S5B) at F= 0.05 pN. Left images are snapshots of dCas9^{alexa549} & ^{SytoxGreen}DNA$_{parS}$ overlay and ParB^{alexa647} signal at time of recruitment (2). Yellow dashed line indicates the position of the dCas9^{alexa549}. Scale bar 30s. Zoomed region (1) represents event where ParB is recruited over the dCas9-roadblock, and continues diffusion over it. The total length of the trace is denoted below. **B)** Quantification of the kymograph data of panel A, that displays the ParB signal in the top (blue, raw data – light blue) and bottom (orange raw data – light orange) part of the DNA, i.e., above and below the Cas9 (dashed yellow line), respectively. (1) represents a crossing event where the intensity increases on the 'no *parS* side'. **C)** Same as in panel A. Zoomed regions (1) and (2) represent two events where ParB is recruited over the dCas9-roadblock, and continues diffusion over it. The total length of the traces are denoted below. **D)** Same as panel B. (1) and (2) represent crossing events that are marked by an intensity increase on the 'no-*parS* side'. **E)** Cartoon representation of the hypothesized ParB-ParB *in-trans* recruitment event.

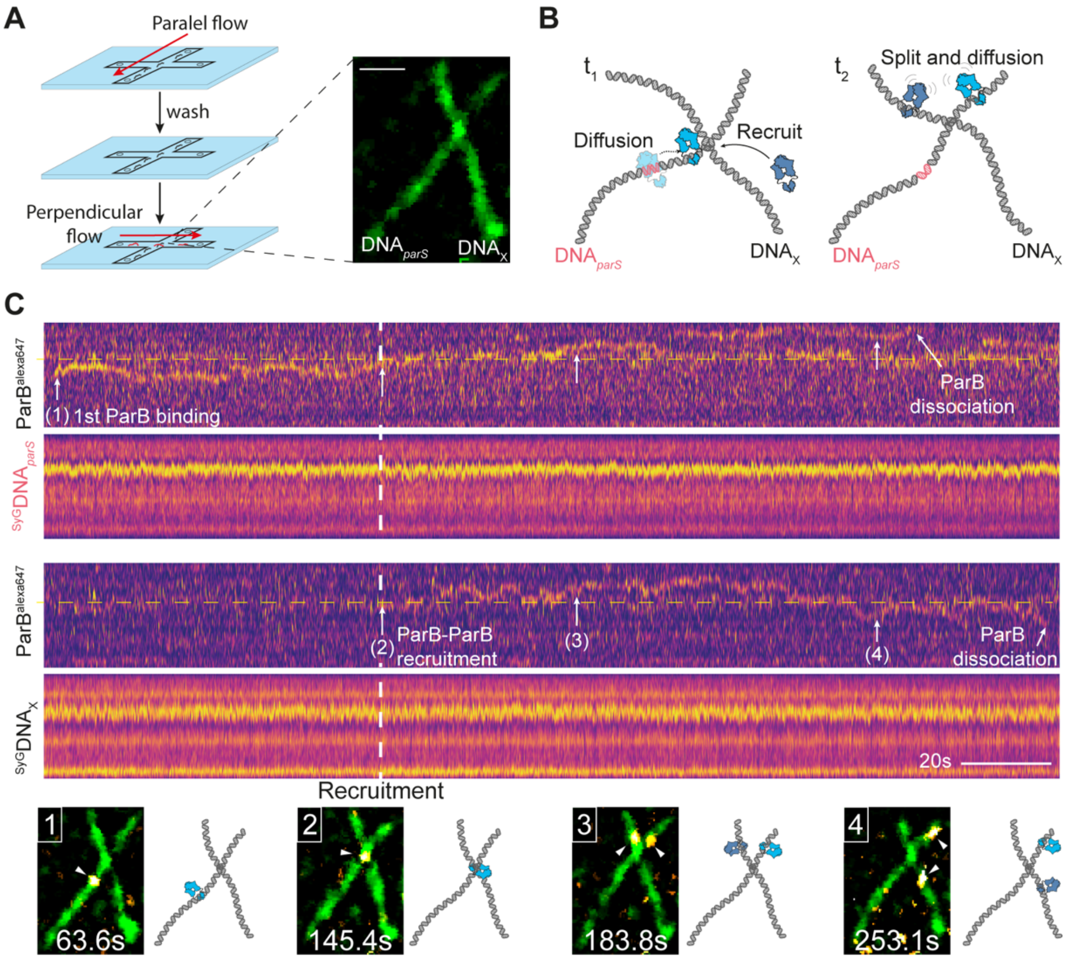

Figure S7. A new ParB dimer can be recruited by a ParB dimer on a different DNA molecule. A) Schematic representation of the experimental setup flow cell and simplified experimental procedure (see Methods). Parallel flow is applied to bind a DNA$_{parS}$ molecule to the surface. After washing, perpendicular flow is applied to bind DNA$_X$ molecules at a large angle compared to the initially bound DNA$_{parS}$. Right: single frame snapshot of the final arrangement of DNA$_{parS}$-DNA$_X$. Scale bar: 2 µm **B)** Cartoon representation of the experiment. *parS*-sequence is indicated in pink **C)** Kymographs for ParB^{alexa647} (top) and DNA stained with SytoxGreen (bottom). Top two kymographs represent the DNA$_{parS}$ signal taken from intensity profiles along the DNA$_{parS}$ molecule. Bottom two kymographs represent DNA$_X$ signal taken from intensity profiles along the DNA$_X$ molecule. Dashed yellow line indicates the approximate junction point. Dashed white vertical line indicates the time when the event occurred where ParB on the DNA$_{parS}$ molecule met the junction point and a new ParB dimer was loaded onto the DNA$_X$ molecule. Bottom row shows single frame snapshots (with corresponding cartoon representations on the side) of the DNA$_{parS}$-DNA$_X$ molecules at timepoints indicated by (1)-(4).

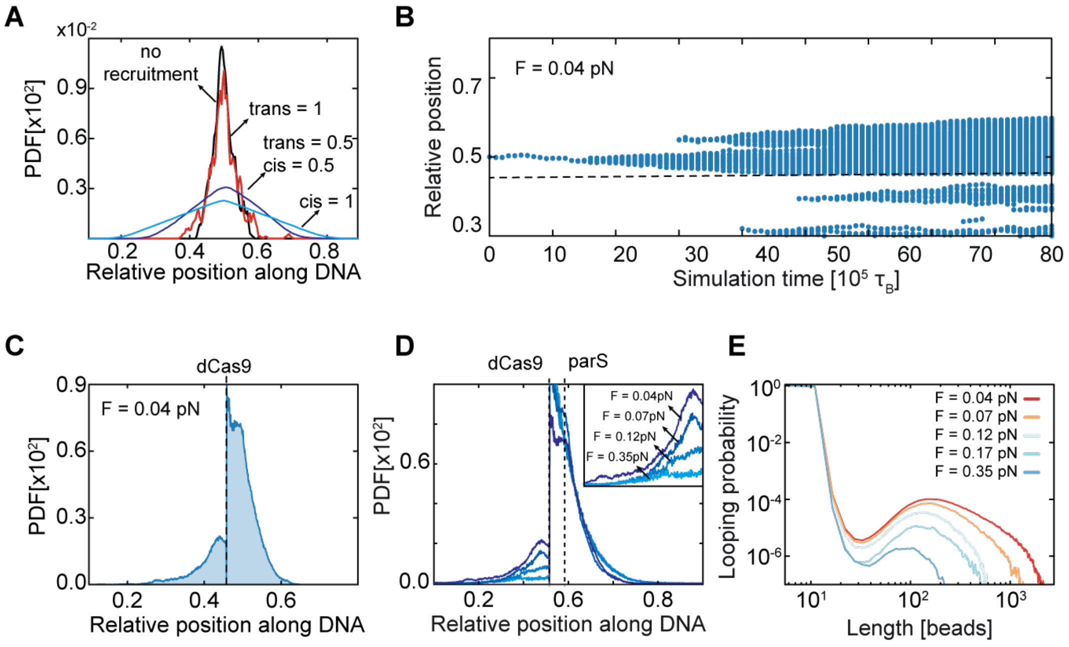

Figure S8. *In-trans* recruitment leads to roadblock-passing via DNA looping. A) ParB spreading with different in-built recruitment ratios (in all cases: *cis*+*trans* = 1, *see Methods*) compared to "no recruitment" scenario, equivalent to clamping and spreading model. **B)** Histogram of ParB positions during simulation experiments (F = 0.04 pN). Dashed line indicates the position of the roadblock particle. **C)** Histogram of cumulated ParB probability density from MD simulations averaged over n=64 simulations represented in panel B. Dashed line indicates the position of the roadblock particle. **D)** Same as C, with overlaid histograms at different forces (tether lengths). **E)** Polymer looping probability as the function of tether length (force to length conversion based on WLC model (*2*)**.**

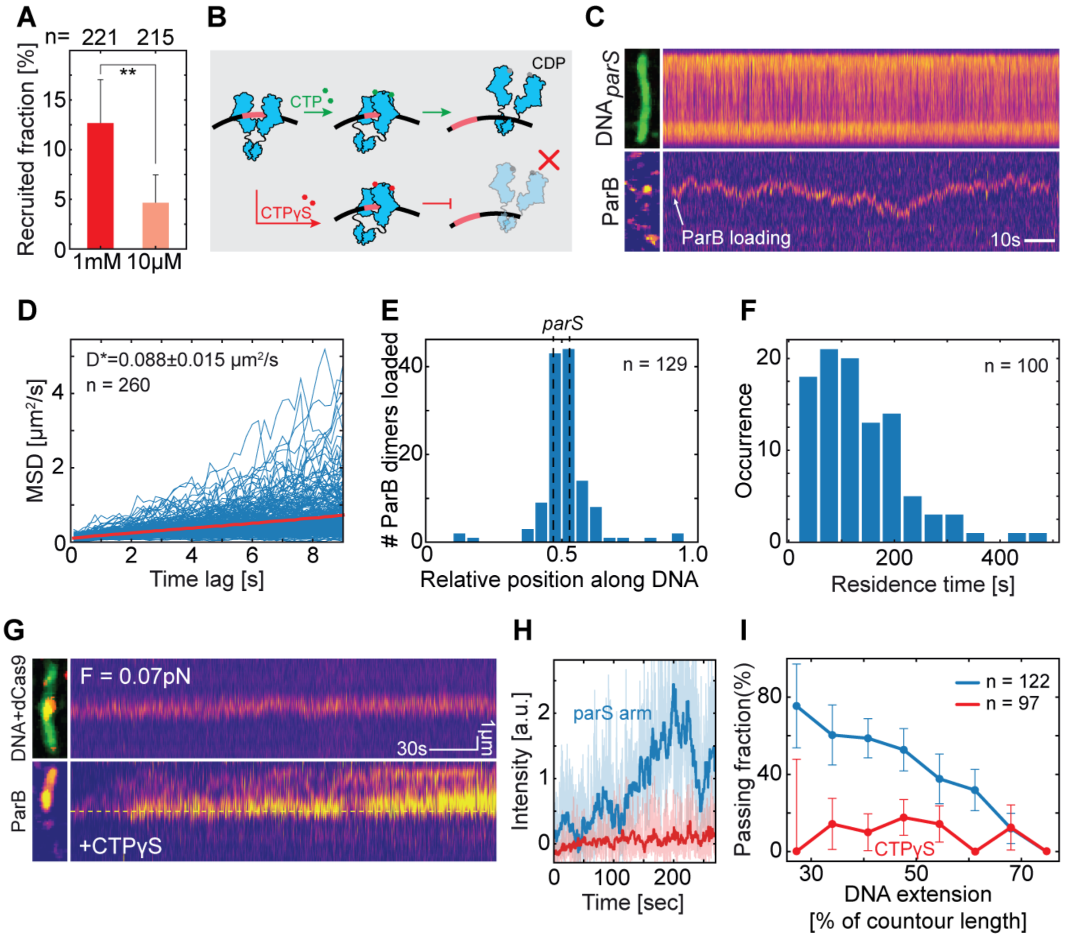

Figure S9. CTPase activity is not required for spreading but is required for ParB-ParB recruitment. A) Recruitment rate in presence of CTP at 1 mM (red) or 10 µM (pink). Error bars represent binominal confidence intervals. Statistical significance calculated using chi-squared test for a binomial distribution, χ^2(n=405) = 8.8072, p<0.003.**B)** Cartoon representation of ParB in the presence of CTP and CTPγS. While CTP allows for hydrolysis and clamp opening, CTPγS largely prevents that and retains ParB in a closed conformation (*3*, *4*). **C)** Kymographs for DNA$_{parS}$ stained with SytoxGreen (top) and ParB^{alexa647} (bottom). Single frame snapshots of the DNA and ParB at the moment of binding, are provided on the left. White arrow indicates ParB loading. Scale bar = 10s. **D)** Mean square displacement of the diffusing ParB molecules loaded at *parS* site in the presence of CTPγS. Apparent diffusion coefficient is D = 0.088±0.015 µm^2/s, n= 260. **E)** Mirrored histogram representing loading position of ParB dimer molecules in the presence of CTPγS relative to the DNA$_{parS}$ ends. *parS* site position is represented by dashed lines. n=129. **F)** Residence times of diffusing ParB molecules after binding the DNA$_{parS}$ in presence of CTPγS (n=100). **G)** Kymograph for dCas9^{alexa549} (top), and ParB^{alexa647} (bottom) for DNA$_{parS}$ at F= 0.07 pN in the presence of CTPγS. Snapshots of dCas9^{alexa549} and ^{SytoxGreen}DNA$_{parS}$ overlay and ParB^{alexa647} signal are provided on the left. Yellow dashed line indicates the position of the dCas9^{alexa549}. **H)** Quantification of the kymograph data of panel F, that displays the ParB signal in the top (blue) and bottom (red) part of the DNA, i.e. above and below the Cas9 (dashed yellow line), respectively. F = 0.07 pN. **I)** Fraction of DNA molecules that exhibit ParB dimers passing the dCas9-roadblock. Blue

and red data are for CTP (blue, n=122) and slowly-hydrolysable CTPγS (red, n=97), respectively(*5*). Error bars represent binomial proportion confidence intervals.

Table S1. Short DNA and RNA sequences used in this study.

Name	Sequence
**Primers**	
JT337	5‘-AGAATAGACCGAGATAGGGTTGAGTG-3‘
JT338	5‘-GGCAGGGTCGGAACAGGAGAG-3‘
JT138	5‘-CTGGCCTTTTGCTCACATGTTCTTTC-3‘
JT139	5‘-CAGATTTAGAAGGCAGATCACCAG-3‘
**Gene blocks**	
parS fragment	5‘-CCAATCACAATCACATCGTATTGGCCTACAATCGCACTTTCACAGGTTCTTTTTTCTTTGTTACACGTGGAACATTCTGATTCAACTGCTGAATTCAAGTGTACAGAGACCCACTGCTTGAGCCTAGAAG-3‘
**RNA constructs**	
crRNA-r2	5‘-GGCATCGGTCGAGGAACTTTCGG-3‘

Table S2: Fit results of the residence time distributions for ParB^{TMR} and ParB^{alexa647}. k_{CTP}: CTP hydrolysis rate, k_{off}: ParB dissociation rate, k_{bl}: bleaching rate, BIC: Bayesian information criterion. The bleaching rate k_{bl} was fixed. Errors represent 68% confidence intervals.

**Model**	**Immediate release**		**Delayed release**	
**Dye**	**TMR**	**Alexa647**	**TMR**	**Alexa647**
k_{bl} **(s^{-1})**	0	$1.75 \cdot 10^{-4}$	0	$1.75 \cdot 10^{-4}$
k_{CTP} **(s^{-1})**	0.0137 ± 0.0005	0.019 ± 0.001	0.022 ± 0.003	0.025 ± 0.003
k_{off} **(s^{-1})**	-	-	0.025 ± 0.005	0.06 ± 0.02
**BIC**	3381.75	3432.92	3362.96	3418.34

Movie S1. Exemplary video of ParB proteins crossing DNA-bound roadblock via ParB-ParB recruitment.

Movie S2. Exemplary video of ParB proteins efficiently blocked by DNA-bound roadblock in presence of CTPγS.

Movie S3. Molecular dynamics simulations of ParB proteins spreading in presence DNA-bound roadblock at low forces.

Movie S4. Molecular dynamics simulations of ParB proteins spreading in presence DNA-bound roadblock at higher forces.
